# Supplementary material for: The burden of post-acute COVID-19 symptoms in a multinational network cohort analysis
Source: Nat Commun. 2023 Nov 17;14:7449. doi: 10.1038/s41467-023-42726-0 (PMC10656441; doi:10.1038/s41467-023-42726-0)
Supplement: Supplementary file 1 — Supplementary Information [file 41467_2023_42726_MOESM1_ESM.pdf]

# SUPPLEMENTARY INFORMATION

## The burden of post-acute COVID-19 symptoms in a multinational network cohort analysis

### Table of Contents

Figure S1: Algorithm to identify post-acute COVID-19 symptoms in routinely collected data ....2
Table S1: Baseline characteristics of the first infections, re-infections, and all negative SARS-CoV-2 test records cohorts ....3
Table S2: Symptom distribution across cohorts (unmatched) - 90 days....5
Table S3: Symptom distribution across cohorts (unmatched) – 28 days ....7
Figure S2: Incidence rates (IR) of COVID-19 and post-acute COVID19 symptoms ....9
Table S4: Incidence rates of COVID-19 and post-acute COVID-19 symptoms ....10
Table S5: Baseline characteristics - COVID-19 infection vs first negative test matched 3:1....13
Table S6: Rate Ratios of post-acute COVID-19 symptoms: COVID-19 infection vs first SARS-CoV-2 negative test - 90 days....15
Table S7: Rate Ratios of post-acute COVID-19 symptoms: COVID-19 infection vs first SARS-CoV-2 negative test - 28 days....17
Table S8: Baseline characteristics: first COVID-19 infection vs re-infections matched 1:3....19
Figure S3: Rate Ratios of post-acute COVID-19 symptoms: COVID-19 infection vs first SARS-CoV-2 negative test - 28 days....21
Table S9: Rate ratios of post-acute COVID-19 symptoms, COVID-19 infection vs all SARS-CoV-2 negative tests - 90 days....22
Figure S4: Rate Ratios of post-acute COVID-19 symptoms, COVID-19 infection vs all SARS-CoV-2 negative tests - 90 days ....24
Table S10: Rate ratio of post-acute COVID-19 symptoms, COVID-19 infection vs all SARS-CoV-2 negative tests - 28 days....25
Figure S5: Rate ratios of post-acute COVID-19 symptoms, COVID-19 infection vs all SARS-CoV-2 negative tests - 28 days....27
Table S11: Rate ratio of post-acute COVID-19 symptoms, first infection vs re-infection - 90 days....28
Table S12: Rate ratios of post-acute COVID-19 symptoms, first infection vs re-infection - 28 days ....30
Figure S6: Rate ratio of post-acute COVID-19 symptoms, first infection vs re-infection - 28 days....32

Figure S1: Algorithm to identify post-acute COVID-19 symptoms in routinely collected data

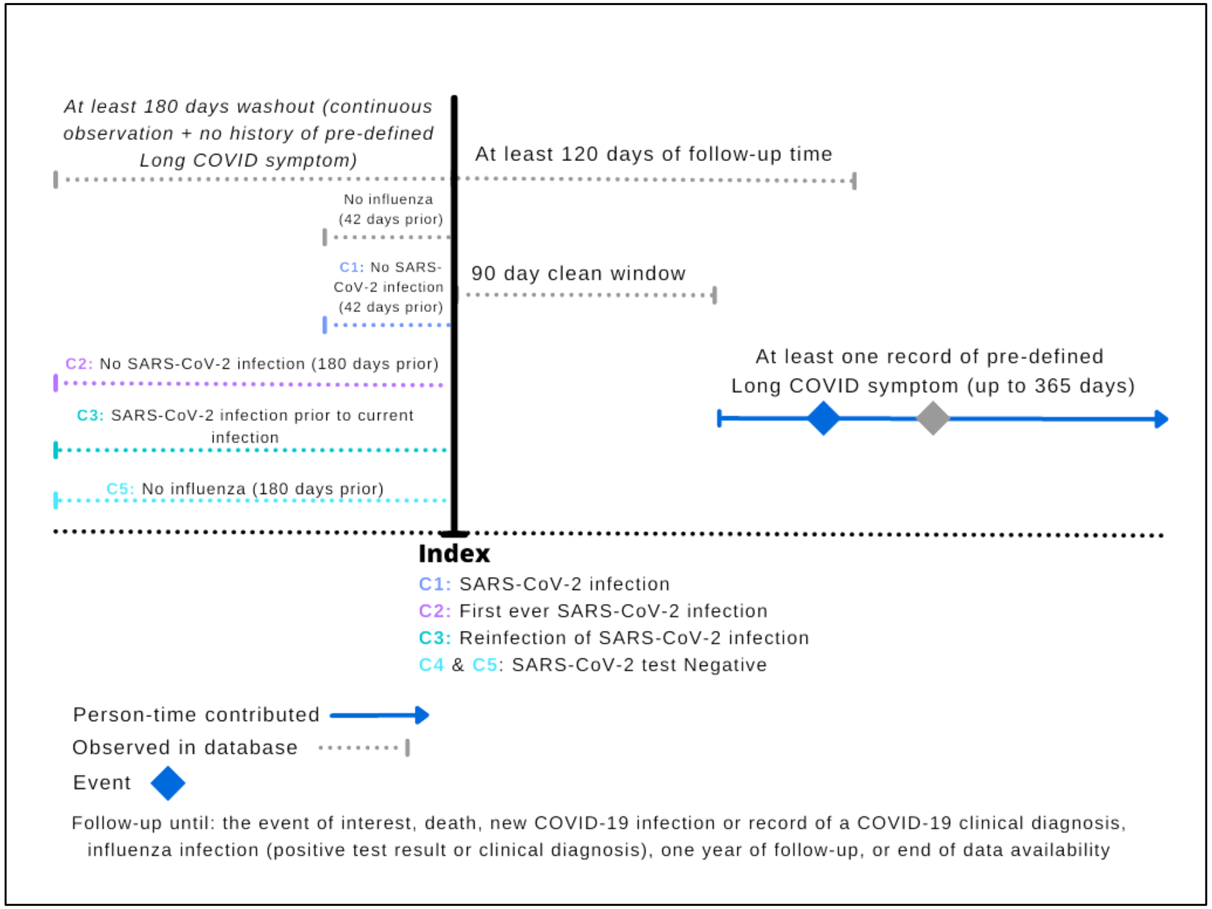

Table S1: Baseline characteristics of the first infections, re-infections, and all negative SARS-CoV-2 test records cohorts

| | SIDIAP | | | CPRD | | |
|---|---|---|---|---|---|---|
| | **First infections** | **Re-infections** | **All SARS-CoV-2 negative tests** | **First infections** | **Re-infections** | **All SARS-CoV-2 negative tests** |
| N | 392,904 | 55,457 | 2,787,432 | 463,936 | 16,965 | 2,012,848 |
| Days of follow-up (median [IQR]) | 349 [251, 365] | 270 [215, 365] | 349 [241, 365] | 365 [308, 365] | 299 [201, 365] | 365 [324, 365] |
| Age (median [IQR]) | 42 [29, 55] | 44 [30, 58] | 47 [34, 62] | 41 [29, 54] | 47 [33, 59] | 41 [30, 54] |
| Age, categories (%) | | | | | | |
| ≤34 | 141,911 (36.1) | 17,977 (32.4) | 739,769 (26.5) | 172,011 (37.1) | 4,620 (27.2) | 712,149 (35.4) |
| 35-49 | 116,891 (29.8) | 16,056 (29.0) | 782,305 (28.1) | 135,258 (29.2) | 4,717 (27.8) | 618,898 (30.7) |
| 50-64 | 78,742 (20.0) | 12,182 (22.0) | 650,411 (23.3) | 107,442 (23.2) | 4,662 (27.5) | 470,049 (23.4) |
| 65-79 | 36,826 (9.4) | 4,382 (7.9) | 432,394 (15.5) | 35,255 (7.6) | 1,949 (11.5) | 158,345 (7.9) |
| ≥80 | 18,534 (4.7) | 4,860 (8.8) | 182,553 (6.5) | 13,970 (3.0) | 1,017 (6.0) | 53,407 (2.7) |
| Sex, male (%) | 176,929 (45.0) | 18,972 (34.2) | 1,246,390 (44.7) | 211,670 (45.6) | 6,499 (38.3) | 853,178 (42.4) |
| Test date period (%) | | | | | | |
| Sep-Dec 2020 | 116,323 (29.6) | 11,096 (20.0) | 907,680 (32.6) | 207,489 (44.7) | 4,168 (24.6) | 1,119,960 (55.6) |
| Jan-Apr 2021 | 120,525 (30.7) | 17,014 (30.7) | 877,404 (31.5) | 178,783 (38.5) | 7,244 (42.7) | 849,653 (42.2) |
| May-Aug 2021 | 136,967 (34.9) | 22,937 (41.4) | 666,648 (23.9) | 69,317 (14.9) | 5,088 (30.0) | 43,040 (2.1) |
| Sep-Dec 2021 | 19,089 (4.9) | 4,410 (8.0) | 335,700 (12.0) | 8,347 (1.8) | 465 (2.7) | 195 (0.0) |
| Wave (%) | | | | | | |
| Alpha | 144,775 (36.8) | 20,384 (36.8) | 1,105,197 (39.6) | 242,997 (52.4) | 9,096 (53.6) | 1,122,116 (55.7) |
| Delta | 150,593 (38.3) | 26,378 (47.6) | 921,001 (33.0) | 75,117 (16.2) | 4,790 (28.2) | 9,980 (0.5) |
| Wild | 97,536 (24.8) | 8,695 (15.7) | 761,234 (27.3) | 145,822 (31.4) | 3,079 (18.1) | 880,752 (43.8) |
| Vaccination status (%) | | | | | | |
| Not vaccinated | 70,304 (17.9) | 9,749 (17.6) | 399,468 (14.3) | 72,905 (15.7) | 2,678 (15.8) | 210,455 (10.5) |
| First dose | 126,784 (32.3) | 22,230 (40.1) | 591,974 (21.2) | 67,142 (14.5) | 4,354 (25.7) | 165,689 (8.2) |
| Two doses | 118,253 (30.1) | 14,529 (26.2) | 756,521 (27.1) | 138,072 (29.8) | 5,274 (31.1) | 653,896 (32.5) |
| Three or more (booster) doses | 77,563 (19.7) | 8,949 (16.1) | 1,039,469 (37.3) | 185,817 (40.1) | 4,659 (27.5) | 982,808 (48.8) |
| COVID-19 PCR test (%) | 211,379 (53.8) | 21,902 (39.5) | 2,787,432 (100.0) | 333,423 (71.9) | 5,081 (29.9) | 2,012,848 (100.0) |

| | | | | | | |
|---|---|---|---|---|---|---|
| Comorbidities (%) | | | | | | |
| Asthma | 29,435 (7.5) | 5,294 (9.5) | 232,144 (8.3) | 76,530 (16.5) | 4,035 (23.8) | 347,009 (17.2) |
| Autoimmune disease | 7,004 (1.8) | 1,422 (2.6) | 67,244 (2.4) | 12,049 (2.6) | 727 (4.3) | 55,379 (2.8) |
| COPD | 9,752 (2.5) | 2,108 (3.8) | 126,395 (4.5) | 8,567 (1.8) | 896 (5.3) | 34,282 (1.7) |
| Dementia | 5,752 (1.5) | 2,625 (4.7) | 41,643 (1.5) | 4,906 (1.1) | 386 (2.3) | 27,819 (1.4) |
| Diabetes | 30,567 (7.8) | 6,042 (10.9) | 283,870 (10.2) | 33,494 (7.2) | 2,142 (12.6) | 127,917 (6.4) |
| Heart disease | 41,061 (10.5) | 8,665 (15.6) | 414,503 (14.9) | 32,599 (7.0) | 2,330 (13.7) | 134,840 (6.7) |
| Cancer | 24,251 (6.2) | 4,855 (8.8) | 278,091 (10.0) | 20,128 (4.3) | 1,131 (6.7) | 92,006 (4.6) |
| Hypertension | 64,156 (16.3) | 12,071 (21.8) | 635,256 (22.8) | 62,514 (13.5) | 3,616 (21.3) | 258,222 (12.8) |
| Renal impairment | 14,885 (3.8) | 3,894 (7.0) | 166,646 (6.0) | 18,621 (4.0) | 1,514 (8.9) | 71,467 (3.6) |

SIDIAP = Sistema d'Informació per al Desenvolupament de la Investigació en Atenció Primària, CPRD = Clinical Practice Research Datalink, IQR = interquartile range, with q25 and q75 provided. COPD = Chronic Obstructive Pulmonary Disease

Table S2: Symptom distribution across cohorts (unmatched) - 90 days

| | SIDIAP | | | | | CPRD | | | | |
|---|---|---|---|---|---|---|---|---|---|---|
| | **COVID-19 infections** | **First infections** | **Re-infections** | **All SARS-CoV-2 negative tests** | **First SARS-CoV-2 negative tests** | **COVID-19 infections** | **First infections** | **Re-infections** | **All SARS-CoV-2 negative tests** | **First SARS-CoV-2 negative tests** |
| N | 448,361 | 392,904 | 55,457 | 2,787,432 | 1,644,166 | 480,901 | 463,936 | 16,965 | 2,012,848 | 1,508,585 |
| Post-acute COVID-19 symptoms, any (%) | 100,989 (22.5) | 84,016 (21.4) | 16,973 (30.6) | 605,318 (21.7) | 350,783 (21.3) | 100,933 (21.0) | 95,652 (20.6) | 5,281 (31.1) | 477,541 (23.7) | 346,553 (23.0) |
| Symptoms (%) | | | | | | | | | | |
| Abdominal pain | 17,512 (3.9) | 14,452 (3.7) | 3,060 (5.5) | 96,956 (3.5) | 56,104 (3.4) | 12,761 (2.7) | 12,099 (2.6) | 662 (3.9) | 62,569 (3.1) | 45,114 (3.0) |
| Allergy | 6,681 (1.5) | 5,564 (1.4) | 1,117 (2.0) | 36,598 (1.3) | 21,999 (1.3) | 4,729 (1.0) | 4,480 (1.0) | 249 (1.5) | 22,530 (1.1) | 16,249 (1.1) |
| Altered smell or taste | 476 (0.1) | 410 (0.1) | 66 (0.1) | 991 (0.0) | 573 (0.0) | 592 (0.1) | 568 (0.1) | 24 (0.1) | 1,104 (0.1) | 785 (0.1) |
| Anxiety | 10,286 (2.3) | 8,486 (2.2) | 1,800 (3.2) | 60,374 (2.2) | 34,200 (2.1) | 14,039 (2.9) | 13,367 (2.9) | 672 (4.0) | 73,665 (3.7) | 52,832 (3.5) |
| Blurred vision | 2,385 (0.5) | 1,944 (0.5) | 441 (0.8) | 13,944 (0.5) | 8,225 (0.5) | 575 (0.1) | 543 (0.1) | 32 (0.2) | 2,697 (0.1) | 1,981 (0.1) |
| Chest pain/ angina | 6,122 (1.4) | 5,019 (1.3) | 1,103 (2.0) | 36,476 (1.3) | 20,657 (1.3) | 6,423 (1.3) | 6,035 (1.3) | 388 (2.3) | 29,488 (1.5) | 21,478 (1.4) |
| Cognitive dysfunction | 1,139 (0.3) | 899 (0.2) | 240 (0.4) | 7,968 (0.3) | 4,511 (0.3) | 355 (0.1) | 322 (0.1) | 33 (0.2) | 1,576 (0.1) | 1,039 (0.1) |
| Cough | 7,301 (1.6) | 5,840 (1.5) | 1,461 (2.6) | 41,738 (1.5) | 22,646 (1.4) | 14,891 (3.1) | 13,920 (3.0) | 971 (5.7) | 70,278 (3.5) | 49,085 (3.3) |
| Depression | 4,348 (1.0) | 3,516 (0.9) | 832 (1.5) | 30,215 (1.1) | 16,637 (1.0) | 13,859 (2.9) | 13,122 (2.8) | 737 (4.3) | 73,552 (3.7) | 53,008 (3.5) |
| Dizziness | 7,702 (1.7) | 6,363 (1.6) | 1,339 (2.4) | 45,433 (1.6) | 26,683 (1.6) | 3,968 (0.8) | 3,754 (0.8) | 214 (1.3) | 18,886 (0.9) | 13,908 (0.9) |
| Dyspnoea | 2,851 (0.6) | 2,297 (0.6) | 554 (1.0) | 17,364 (0.6) | 9,473 (0.6) | 9,069 (1.9) | 8,474 (1.8) | 595 (3.5) | 36,660 (1.8) | 27,294 (1.8) |
| Fatigue or malaise | 6,820 (1.5) | 5,566 (1.4) | 1,254 (2.3) | 37,867 (1.4) | 21,609 (1.3) | 7,938 (1.7) | 7,476 (1.6) | 462 (2.7) | 33,964 (1.7) | 24,279 (1.6) |
| Gastrointestinal issues | 12,415 (2.8) | 10,159 (2.6) | 2,256 (4.1) | 80,601 (2.9) | 45,485 (2.8) | 12,094 (2.5) | 11,374 (2.5) | 720 (4.2) | 59,133 (2.9) | 42,501 (2.8) |
| Headache | 8,255 (1.8) | 6,772 (1.7) | 1,483 (2.7) | 45,165 (1.6) | 25,479 (1.5) | 8,756 (1.8) | 8,318 (1.8) | 438 (2.6) | 44,279 (2.2) | 31,578 (2.1) |
| Intermittent fever | 5,509 (1.2) | 4,405 (1.1) | 1,104 (2.0) | 32,023 (1.1) | 17,443 (1.1) | 465 (0.1) | 419 (0.1) | 46 (0.3) | 2,132 (0.1) | 1,456 (0.1) |
| Joint pain | 17,618 (3.9) | 14,586 (3.7) | 3,032 (5.5) | 104,226 (3.7) | 62,057 (3.8) | 12,654 (2.6) | 12,001 (2.6) | 653 (3.8) | 58,939 (2.9) | 43,728 (2.9) |
| Memory issues | 832 (0.2) | 692 (0.2) | 140 (0.3) | 5,961 (0.2) | 3,498 (0.2) | 619 (0.1) | 565 (0.1) | 54 (0.3) | 2,296 (0.1) | 1,738 (0.1) |
| Menstrual problems | 2,567 (0.6) | 2,090 (0.5) | 477 (0.9) | 11,661 (0.4) | 6,680 (0.4) | 2,856 (0.6) | 2,758 (0.6) | 98 (0.6) | 14,240 (0.7) | 10,179 (0.7) |
| Muscle spasms and pain | 562 (0.1) | 455 (0.1) | 107 (0.2) | 4,290 (0.2) | 2,565 (0.2) | 276 (0.1) | 261 (0.1) | 15 (0.1) | 1,351 (0.1) | 1,037 (0.1) |

| | | | | | | | | | | |
|---|---|---|---|---|---|---|---|---|---|---|
| Neuralgia | 4,661 (1.0) | 3,817 (1.0) | 844 (1.5) | 29,216 (1.0) | 17,029 (1.0) | 2,401 (0.5) | 2,281 (0.5) | 120 (0.7) | 11,369 (0.6) | 8,441 (0.6) |
| Pins and needles sensation | 1,703 (0.4) | 1,382 (0.4) | 321 (0.6) | 10,613 (0.4) | 6,223 (0.4) | 925 (0.2) | 863 (0.2) | 62 (0.4) | 4,524 (0.2) | 3,293 (0.2) |
| Post exertional fatigue | 0 | 0 | 0 | 0 | 0 | 0 | 0 | 0 | 0 | 0 |
| Sleep disorder | 4,788 (1.1) | 3,935 (1.0) | 853 (1.5) | 34,882 (1.3) | 19,806 (1.2) | 3,885 (0.8) | 3,633 (0.8) | 252 (1.5) | 18,997 (0.9) | 13,743 (0.9) |
| Tachycardia | 1,997 (0.4) | 1,659 (0.4) | 338 (0.6) | 12,002 (0.4) | 6,726 (0.4) | 4,409 (0.9) | 4,149 (0.9) | 260 (1.5) | 19,828 (1.0) | 14,464 (1.0) |
| Tinnitus hearing problems | 2,893 (0.6) | 2,373 (0.6) | 520 (0.9) | 19,450 (0.7) | 11,548 (0.7) | 3,264 (0.7) | 3,084 (0.7) | 180 (1.1) | 15,211 (0.8) | 11,146 (0.7) |
| Number of symptoms (%) | | | | | | | | | | |
| 0 | 347,372 (77.5) | 308,888 (78.6) | 38,484 (69.4) | 2,182,114 (78.3) | 1,293,383 (78.7) | 379,968 (79.0) | 368,284 (79.4) | 11,684 (68.9) | 1,535,307 (76.3) | 1,162,032 (77.0) |
| 1 | 74,501 (16.6) | 62,880 (16.0) | 11,621 (21.0) | 450,240 (16.2) | 263,676 (16.0) | 71,026 (14.8) | 67,528 (14.6) | 3,498 (20.6) | 330,191 (16.4) | 241,045 (16.0) |
| 2 | 19,253 (4.3) | 15,585 (4.0) | 3,668 (6.6) | 114,073 (4.1) | 64,822 (3.9) | 22,077 (4.6) | 20,882 (4.5) | 1,195 (7.0) | 107,339 (5.3) | 77,269 (5.1) |
| 3 | 5,214 (1.2) | 4,063 (1.0) | 1,151 (2.1) | 30,049 (1.1) | 16,511 (1.0) | 5,604 (1.2) | 5,207 (1.1) | 397 (2.3) | 29,403 (1.5) | 20,793 (1.4) |
| 4 | 1,507 (0.3) | 1,117 (0.3) | 390 (0.7) | 8,162 (0.3) | 4,327 (0.3) | 1,589 (0.3) | 1,455 (0.3) | 134 (0.8) | 7,843 (0.4) | 5,505 (0.4) |
| 5 | 379 (0.1) | 278 (0.1) | 101 (0.2) | 2,128 (0.1) | 1,094 (0.1) | 457 (0.1) | 423 (0.1) | 34 (0.2) | 2,065 (0.1) | 1,463 (0.1) |
| 6 | 104 (0.0) | 74 (0.0) | 30 (0.1) | 512 (0.0) | 267 (0.0) | 117 (0.0) | 103 (0.0) | 14 (0.1) | 486 (0.0) | 339 (0.0) |
| 7 | 24 (0.0) | 14 (0.0) | 10 (0.0) | 119 (0.0) | 70 (0.0) | 43 (0.0) | 37 (0.0) | 6 (0.0) | 159 (0.0) | 104 (0.0) |
| 8 | <5 | <5 | <5 | 29 (0.0) | 13 (0.0) | 16 (0.0) | 14 (0.0) | <5 | 38 (0.0) | 23 (0.0) |
| 9 | <5 | <5 | <5 | <5 | <5 | <5 | <5 | <5 | 13 (0.0) | 9 (0.0) |
| 10 | <5 | <5 | <5 | <5 | <5 | <5 | <5 | <5 | <5 | <5 |
| Days elapsed since index date and record of symptom (median [IQR]) | 177 [127, 244] | 181 [129, 247] | 161 [119, 226] | 180 [129, 251] | 188 [134, 261] | 183 [128, 260] | 184 [128, 262] | 161 [117, 224] | 190 [133, 261] | 193 [134, 265] |

SIDIAP = Sistema d'Informació per al Desenvolupament de la Investigació en Atenció Primària, CPRD = Clinical Practice Research Datalink, IQR = interquartile range, with q25 and q75 provided.

Table S3: Symptom distribution across cohorts (unmatched) – 28 days

| | SIDIAP | | | | | CPRD | | | | |
|---|---|---|---|---|---|---|---|---|---|---|
| | **COVID-19 infection** | **First infections** | **Re-infections** | **All SARS-CoV-2 negative tests** | **First SARS-CoV-2 negative tests** | **COVID-19 infections** | **First infections** | **Re-infections** | **All SARS-CoV-2 negative tests** | **First SARS-CoV-2 negative tests** |
| N | 448,361 | 392,904 | 55,457 | 2,787,432 | 1,644,166 | 480,901 | 463,936 | 16,965 | 2,012,848 | 1,508,585 |
| Post-acute COVID-19 symptoms, any (%) | 125,619 (28.0) | 103,868 (26.4) | 21,751 (39.2) | 752,040 (27.0) | 427,988 (26.0) | 120,986 (25.2) | 114,545 (24.7) | 6,441 (38.0) | 552,829 (27.5) | 399,722 (26.5) |
| Symptoms (%) | | | | | | | | | | |
| Abdominal pain | 23,000 (5.1) | 18,707 (4.8) | 4,293 (7.7) | 125,916 (4.5) | 71,152 (4.3) | 15,949 (3.3) | 15,092 (3.3) | 857 (5.1) | 76,559 (3.8) | 54,924 (3.6) |
| Allergy | 8,960 (2.0) | 7,322 (1.9) | 1,638 (3.0) | 48,166 (1.7) | 28,006 (1.7) | 5,725 (1.2) | 5,397 (1.2) | 328 (1.9) | 27,272 (1.4) | 19,406 (1.3) |
| Altered smell or taste | 611 (0.1) | 520 (0.1) | 91 (0.2) | 1,356 (0.0) | 786 (0.0) | 770 (0.2) | 734 (0.2) | 36 (0.2) | 1,299 (0.1) | 932 (0.1) |
| Anxiety | 13,651 (3.0) | 11,101 (2.8) | 2,550 (4.6) | 78,926 (2.8) | 43,627 (2.7) | 17,053 (3.5) | 16,219 (3.5) | 834 (4.9) | 87,177 (4.3) | 62,212 (4.1) |
| Blurred vision | 3,217 (0.7) | 2,594 (0.7) | 623 (1.1) | 18,119 (0.7) | 10,412 (0.6) | 748 (0.2) | 701 (0.2) | 47 (0.3) | 3,344 (0.2) | 2,420 (0.2) |
| Chest pain or angina | 8,199 (1.8) | 6,620 (1.7) | 1,579 (2.8) | 47,467 (1.7) | 26,327 (1.6) | 8,366 (1.7) | 7,835 (1.7) | 531 (3.1) | 36,253 (1.8) | 26,283 (1.7) |
| Cognitive dysfunction | 1,511 (0.3) | 1,185 (0.3) | 326 (0.6) | 10,567 (0.4) | 5,876 (0.4) | 451 (0.1) | 408 (0.1) | 43 (0.3) | 1,912 (0.1) | 1,239 (0.1) |
| Cough | 9,533 (2.1) | 7,555 (1.9) | 1,978 (3.6) | 53,074 (1.9) | 28,256 (1.7) | 18,183 (3.8) | 16,977 (3.7) | 1,206 (7.1) | 81,561 (4.1) | 57,089 (3.8) |
| Depression | 5,594 (1.2) | 4,466 (1.1) | 1,128 (2.0) | 39,061 (1.4) | 20,921 (1.3) | 16,572 (3.4) | 15,667 (3.4) | 905 (5.3) | 86,184 (4.3) | 61,922 (4.1) |
| Dizziness | 10,142 (2.3) | 8,260 (2.1) | 1,882 (3.4) | 58,754 (2.1) | 33,645 (2.0) | 4,974 (1.0) | 4,694 (1.0) | 280 (1.7) | 23,113 (1.1) | 16,898 (1.1) |
| Dyspnoea | 4,107 (0.9) | 3,285 (0.8) | 822 (1.5) | 22,972 (0.8) | 12,307 (0.7) | 12,020 (2.5) | 11,179 (2.4) | 841 (5.0) | 44,549 (2.2) | 33,042 (2.2) |
| Fatigue or malaise | 9,281 (2.1) | 7,514 (1.9) | 1,767 (3.2) | 49,447 (1.8) | 27,331 (1.7) | 10,144 (2.1) | 9,525 (2.1) | 619 (3.6) | 41,261 (2.0) | 29,270 (1.9) |
| Gastrointestinal issues | 16,360 (3.6) | 13,252 (3.4) | 3,108 (5.6) | 106,547 (3.8) | 58,963 (3.6) | 15,244 (3.2) | 14,297 (3.1) | 947 (5.6) | 72,063 (3.6) | 51,585 (3.4) |
| Headache | 10,801 (2.4) | 8,802 (2.2) | 1,999 (3.6) | 58,918 (2.1) | 32,538 (2.0) | 10,894 (2.3) | 10,327 (2.2) | 567 (3.3) | 54,415 (2.7) | 38,440 (2.5) |
| Intermittent fever | 7,319 (1.6) | 5,758 (1.5) | 1,561 (2.8) | 43,231 (1.6) | 22,806 (1.4) | 598 (0.1) | 537 (0.1) | 61 (0.4) | 2,637 (0.1) | 1,790 (0.1) |
| Joint pain | 23,075 (5.1) | 18,901 (4.8) | 4,174 (7.5) | 134,154 (4.8) | 78,137 (4.8) | 15,327 (3.2) | 14,503 (3.1) | 824 (4.9) | 69,868 (3.5) | 51,555 (3.4) |
| Memory issues | 1,134 (0.3) | 920 (0.2) | 214 (0.4) | 7,559 (0.3) | 4,320 (0.3) | 799 (0.2) | 726 (0.2) | 73 (0.4) | 2,724 (0.1) | 2,036 (0.1) |
| Menstrual problems | 3,418 (0.8) | 2,770 (0.7) | 648 (1.2) | 15,438 (0.6) | 8,648 (0.5) | 3,666 (0.8) | 3,529 (0.8) | 137 (0.8) | 17,524 (0.9) | 12,410 (0.8) |
| Muscle spasms and pain | 738 (0.2) | 591 (0.2) | 147 (0.3) | 5,550 (0.2) | 3,242 (0.2) | 353 (0.1) | 336 (0.1) | 17 (0.1) | 1,598 (0.1) | 1,221 (0.1) |

| | | | | | | | | | | |
|---|---|---|---|---|---|---|---|---|---|---|
| Neuralgia | 6,098 (1.4) | 4,932 (1.3) | 1,166 (2.1) | 37,924 (1.4) | 21,583 (1.3) | 2,921 (0.6) | 2,773 (0.6) | 148 (0.9) | 13,590 (0.7) | 10,051 (0.7) |
| Pins/needles sensation | 2,263 (0.5) | 1,825 (0.5) | 438 (0.8) | 13,728 (0.5) | 7,816 (0.5) | 1,199 (0.2) | 1,117 (0.2) | 82 (0.5) | 5,565 (0.3) | 4,025 (0.3) |
| Post exertional fatigue | 0 | 0 | 0 | 0 | 0 | 0 | 0 | 0 | 0 | 0 |
| Sleep disorder | 6,461 (1.4) | 5,231 (1.3) | 1,230 (2.2) | 45,983 (1.6) | 25,529 (1.6) | 5,106 (1.1) | 4,775 (1.0) | 331 (2.0) | 23,885 (1.2) | 17,198 (1.1) |
| Tachycardia | 2,716 (0.6) | 2,236 (0.6) | 480 (0.9) | 16,014 (0.6) | 8,796 (0.5) | 5,677 (1.2) | 5,331 (1.1) | 346 (2.0) | 23,950 (1.2) | 17,305 (1.1) |
| Tinnitus hearing problems | 3,825 (0.9) | 3,113 (0.8) | 712 (1.3) | 25,230 (0.9) | 14,684 (0.9) | 4,051 (0.8) | 3,817 (0.8) | 234 (1.4) | 18,387 (0.9) | 13,409 (0.9) |
| Number of symptoms | | | | | | | | | | |
| 0 | 322,742 (72.0) | 289,036 (73.6) | 33,706 (60.8) | 2,035,392 (73.0) | 1,216,178 (74.0) | 359,915 (74.8) | 349,391 (75.3) | 10,524 (62.0) | 1,460,019 (72.5) | 1,108,863 (73.5) |
| 1 | 86,623 (19.3) | 73,223 (18.6) | 13,400 (24.2) | 531,048 (19.1) | 307,139 (18.7) | 81,724 (17.0) | 77,755 (16.8) | 3,969 (23.4) | 366,852 (18.2) | 267,240 (17.7) |
| 2 | 26,864 (6.0) | 21,515 (5.5) | 5,349 (9.6) | 156,118 (5.6) | 86,940 (5.3) | 27,790 (5.8) | 26,208 (5.6) | 1,582 (9.3) | 130,466 (6.5) | 93,539 (6.2) |
| 3 | 8,383 (1.9) | 6,392 (1.6) | 1,991 (3.6) | 45,855 (1.6) | 24,405 (1.5) | 7,962 (1.7) | 7,394 (1.6) | 568 (3.3) | 39,427 (2.0) | 27,722 (1.8) |
| 4 | 2,654 (0.6) | 1,949 (0.5) | 705 (1.3) | 13,674 (0.5) | 6,933 (0.4) | 2,406 (0.5) | 2,195 (0.5) | 211 (1.2) | 11,553 (0.6) | 8,093 (0.5) |
| 5 | 779 (0.2) | 565 (0.1) | 214 (0.4) | 3,949 (0.1) | 1,893 (0.1) | 796 (0.2) | 719 (0.2) | 77 (0.5) | 3,278 (0.2) | 2,279 (0.2) |
| 6 | 242 (0.1) | 179 (0.0) | 63 (0.1) | 1,050 (0.0) | 506 (0.0) | 206 (0.0) | 185 (0.0) | 21 (0.1) | 885 (0.0) | 614 (0.0) |
| 7 | 48 (0.0) | 29 (0.0) | 19 (0.0) | 276 (0.0) | 142 (0.0) | 66 (0.0) | 61 (0.0) | 5 (0.0) | 268 (0.0) | 175 (0.0) |
| 8 | 19 (0.0) | 11 (0.0) | 8 (0.0) | 56 (0.0) | 25 (0.0) | 29 (0.0) | 23 (0.0) | 6 (0.0) | 73 (0.0) | 44 (0.0) |
| 9 | 7 (0.0) | 5 (0.0) | <5 | 9 (0.0) | <5 | 5 (0.0) | <5 | <5 | 20 (0.0) | 12 (0.0) |
| 10 | <5 | <5 | <5 | 5 (0.0) | <5 | <5 | <5 | <5 | <5 | <5 |
| Days elapsed since index date and record of symptom (median [IQR]) | 131 [73, 212] | 135 [77, 217] | 111 [62, 186] | 136 [77, 219] | 146 [82, 231] | 139 [75, 231] | 141 [76, 233] | 115 [64, 191] | 152 [82, 238] | 155 [84, 243] |

SIDIAP = Sistema d'Informació per al Desenvolupament de la Investigació en Atenció Primària, CPRD = Clinical Practice Research Datalink, IQR = interquartile range, with q25 and q75 provided.

Figure S2: Incidence rates (IR) of COVID-19 and post-acute COVID19 symptoms

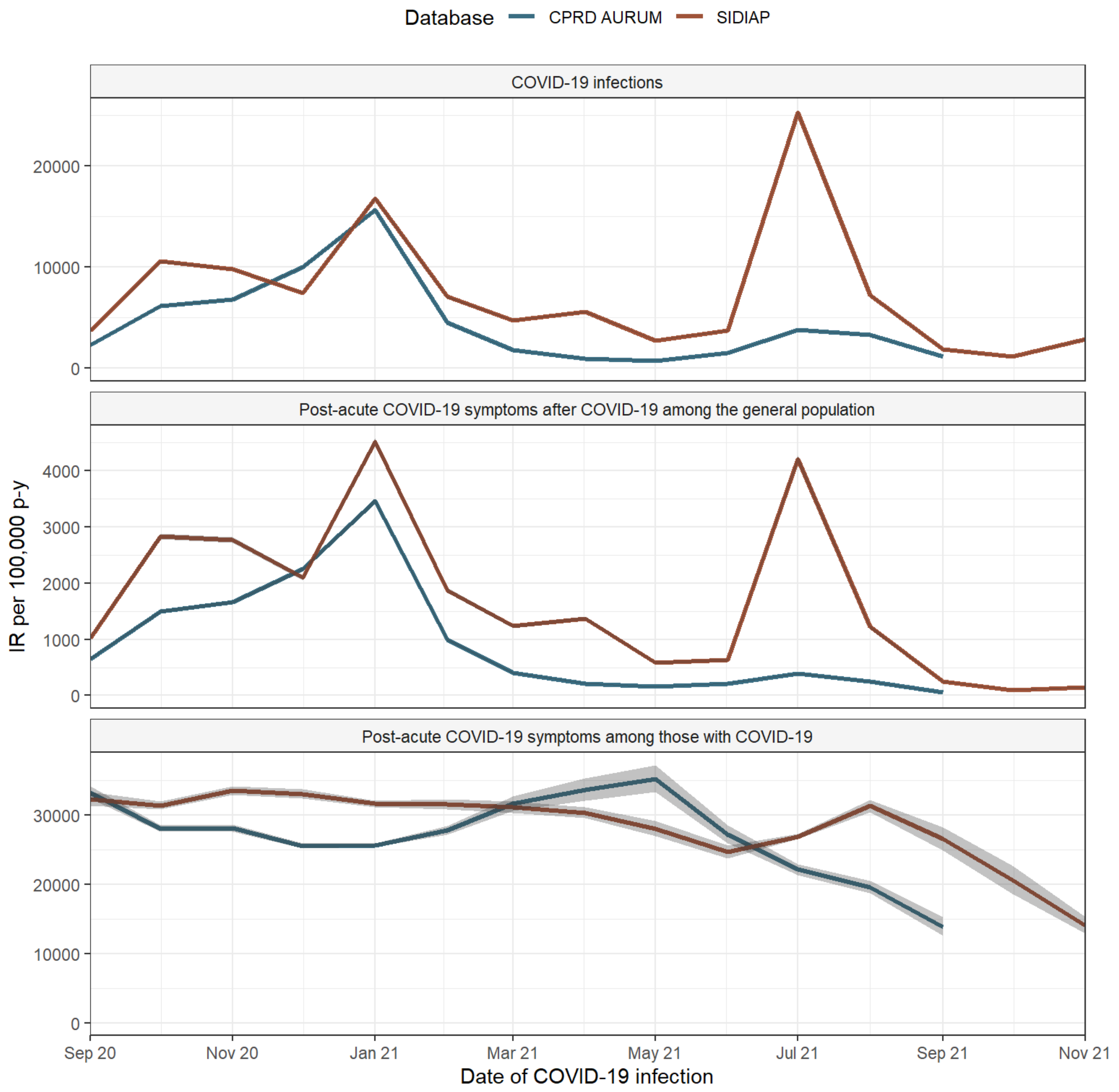


SIDIAP = Sistema d'Informació per al Desenvolupament de la Investigació en Atenció Primària, CPRD = Clinical Practice Research Datalink, IR = incidence rate, p-y = person years

Table S4: Incidence rates of COVID-19 and post-acute COVID-19 symptoms

| Database | Cohort | COVID-19 test date | Number of events | Person-years | Incidence Rate |
|---|---|---|---|---|---|
| CPRD AURUM | COVID-19 infections | 2020-09-01 | 19357 | 832348 | 2325.6 [2292.9-2358.6] |
| | | 2020-10-01 | 52467 | 851056 | 6164.9 [6112.3-6217.9] |
| | | 2020-11-01 | 55491 | 816559 | 6795.7 [6739.3-6852.5] |
| | | 2020-12-01 | 84342 | 840338 | 10036.7 [9969.1-10104.6] |
| | | 2021-01-01 | 130083 | 830063 | 15671.5 [15586.4-15756.9] |
| | | 2021-02-01 | 33511 | 744573 | 4500.7 [4452.6-4549.1] |
| | | 2021-03-01 | 14718 | 824081 | 1786.0 [1757.3-1815.1] |
| | | 2021-04-01 | 7715 | 791775 | 974.4 [952.8-996.4] |
| | | 2021-05-01 | 6124 | 811628 | 754.5 [735.8-773.7] |
| | | 2021-06-01 | 11790 | 779278 | 1512.9 [1485.8-1540.5] |
| | | 2021-07-01 | 30524 | 797498 | 3827.5 [3784.7-3870.7] |
| | | 2021-08-01 | 25967 | 789897 | 3287.4 [3247.5-3327.6] |
| | | 2021-09-01 | 8812 | 758802 | 1161.3 [1137.2-1185.8] |
| CPRD AURUM | Post-acute COVID-19 symptoms after COVID-19 among the general population | 2020-09-01 | 5425 | 832799 | 651.4 [634.2-669.0] |
| | | 2020-10-01 | 12773 | 853537 | 1496.5 [1470.6-1522.7] |
| | | 2020-11-01 | 13607 | 821316 | 1656.7 [1629.0-1684.8] |
| | | 2020-12-01 | 19012 | 845366 | 2249.0 [2217.1-2281.2] |
| | | 2021-01-01 | 29032 | 840112 | 3455.7 [3416.1-3495.7] |
| | | 2021-02-01 | 7465 | 752486 | 992.0 [969.7-1014.8] |
| | | 2021-03-01 | 3381 | 826893 | 408.9 [395.2-422.9] |
| | | 2021-04-01 | 1715 | 792887 | 216.3 [206.2-226.8] |
| | | 2021-05-01 | 1281 | 812320 | 157.7 [149.2-166.6] |
| | | 2021-06-01 | 1692 | 779937 | 216.9 [206.7-227.5] |
| | | 2021-07-01 | 3148 | 799368 | 393.8 [380.2-407.8] |
| | | 2021-08-01 | 1987 | 792893 | 250.6 [239.7-261.9] |
| | | 2021-09-01 | 415 | 761114 | 54.5 [49.4-60.0] |
| CPRD AURUM | Post-acute COVID-19 symptoms among those with COVID-19 | 2020-09-01 | 5425 | 16324 | 33234.3 [32355.7-34130.6] |
| | | 2020-10-01 | 12773 | 45547 | 28043.6 [27559.4-28534.2] |
| | | 2020-11-01 | 13607 | 48418 | 28103.0 [27632.7-28579.2] |
| | | 2020-12-01 | 19012 | 74416 | 25548.1 [25186.3-25913.9] |
| | | 2021-01-01 | 29032 | 113212 | 25643.9 [25349.8-25940.6] |
| | | 2021-02-01 | 7465 | 26850 | 27802.3 [27175.2-28440.3] |
| | | 2021-03-01 | 3381 | 10686 | 31640.9 [30583.3-32725.8] |
| | | 2021-04-01 | 1715 | 5097 | 33647.2 [32073.4-35278.2] |
| | | 2021-05-01 | 1281 | 3641 | 35182.2 [33281.7-37162.9] |
| | | 2021-06-01 | 1692 | 6225 | 27182.0 [25902.1-28508.8] |
| | | 2021-07-01 | 3148 | 14230 | 22122.0 [21355.9-22908.6] |
| | | 2021-08-01 | 1987 | 10144 | 19588.3 [18736.4-20468.9] |
| | | 2021-09-01 | 415 | 2990 | 13879.6 [12576.2-15281.4] |

| Database | Cohort | COVID-19 test date | Number of events | Person-years | Incidence Rate |
|---|---|---|---|---|---|
| SIDIAP | COVID-19 infections | 2020-09-01 | 14852 | 398042 | 3731.3 [3671.5-3791.8] |
| | | 2020-10-01 | 43306 | 409365 | 10578.8 [10479.4-10678.9] |
| | | 2020-11-01 | 38494 | 392832 | 9799.1 [9701.4-9897.5] |
| | | 2020-12-01 | 30326 | 406983 | 7451.4 [7367.8-7535.8] |
| | | 2021-01-01 | 67962 | 404780 | 16789.8 [16663.8-16916.6] |
| | | 2021-02-01 | 25756 | 364265 | 7070.7 [6984.6-7157.6] |
| | | 2021-03-01 | 19270 | 406898 | 4735.8 [4669.2-4803.2] |
| | | 2021-04-01 | 22032 | 394595 | 5583.4 [5510.0-5657.7] |
| | | 2021-05-01 | 11258 | 408125 | 2758.5 [2707.7-2809.9] |
| | | 2021-06-01 | 14783 | 396002 | 3733.1 [3673.1-3793.7] |
| | | 2021-07-01 | 102421 | 404740 | 25305.4 [25150.7-25460.9] |
| | | 2021-08-01 | 29020 | 401964 | 7219.5 [7136.7-7303.1] |
| | | 2021-09-01 | 7442 | 394947 | 1884.3 [1841.7-1927.6] |
| | | 2021-10-01 | 4657 | 409202 | 1138.1 [1105.6-1171.2] |
| | | 2021-11-01 | 11355 | 395327 | 2872.3 [2819.7-2925.6] |
| SIDIAP | Post-acute COVID-19 symptoms after COVID-19 among the general population | 2020-09-01 | 4066 | 398479 | 1020.4 [989.3-1052.2] |
| | | 2020-10-01 | 11630 | 411031 | 2829.5 [2778.3-2881.4] |
| | | 2020-11-01 | 10975 | 396654 | 2766.9 [2715.4-2819.2] |
| | | 2020-12-01 | 8585 | 409744 | 2095.2 [2051.1-2140.0] |
| | | 2021-01-01 | 18414 | 408539 | 4507.3 [4442.4-4572.9] |
| | | 2021-02-01 | 6888 | 368567 | 1868.9 [1825.0-1913.5] |
| | | 2021-03-01 | 5042 | 409072 | 1232.5 [1198.8-1267.0] |
| | | 2021-04-01 | 5437 | 396282 | 1372.0 [1335.8-1409.0] |
| | | 2021-05-01 | 2396 | 409811 | 584.7 [561.5-608.5] |
| | | 2021-06-01 | 2498 | 396956 | 629.3 [604.9-654.5] |
| | | 2021-07-01 | 17181 | 409502 | 4195.6 [4133.1-4258.8] |
| | | 2021-08-01 | 4989 | 409077 | 1219.6 [1186.0-1253.9] |
| | | 2021-09-01 | 986 | 397041 | 248.3 [233.1-264.3] |
| | | 2021-10-01 | 417 | 409884 | 101.7 [92.2-112.0] |
| | | 2021-11-01 | 565 | 396007 | 142.7 [131.2-154.9] |
| SIDIAP | Post-acute COVID-19 symptoms among those with COVID-19 | 2020-09-01 | 4085 | 12655 | 32280.0 [31297.7-33285.4] |
| | | 2020-10-01 | 11697 | 37305 | 31355.2 [30789.6-31928.7] |
| | | 2020-11-01 | 11012 | 32876 | 33495.3 [32872.6-34126.9] |
| | | 2020-12-01 | 8588 | 25978 | 33058.9 [32363.3-33765.6] |
| | | 2021-01-01 | 18668 | 59026 | 31627.0 [31174.9-32084.0] |
| | | 2021-02-01 | 7008 | 22194 | 31576.3 [30841.3-32324.4] |
| | | 2021-03-01 | 5103 | 16403 | 31110.6 [30262.8-31976.1] |
| | | 2021-04-01 | 5507 | 18154 | 30334.3 [29538.4-31146.3] |
| | | 2021-05-01 | 2426 | 8649 | 28050.8 [26945.6-29189.7] |
| | | 2021-06-01 | 2556 | 10358 | 24676.4 [23729.0-25652.0] |
| | | 2021-07-01 | 17352 | 64526 | 26891.3 [26492.7-27294.4] |
| | | 2021-08-01 | 5018 | 16036 | 31292.6 [30432.7-32170.7] |

| Database | Cohort | COVID-19 test date | Number of events | Person-years | Incidence Rate |
|---|---|---|---|---|---|
| | | 2021-09-01 | 987 | 3718 | 26546.3 [24915.8-28255.5] |
| | | 2021-10-01 | 417 | 2027 | 20572.6 [18645.2-22645.2] |
| | | 2021-11-01 | 565 | 4024 | 14041.9 [12907.8-15249.0] |

SIDIAP = Sistema d'Informació per al Desenvolupament de la Investigació en Atenció Primària, CPRD = Clinical Practice Research Datalink, IR = incidence rate, p-y = person years, 95% CI = 95% confidence intervals

Table S5: Baseline characteristics - COVID-19 infection vs first negative test matched 3:1

| | SIDIAP | | | CPRD | | |
|---|---|---|---|---|---|---|
| | **COVID-19 infection** | **First SARS-CoV-2 negative test** | **SMD** | **COVID-19 infections** | **First SARS-CoV-2 negative tests** | **SMD** |
| N | 229,086 | 591,145 | | 332,276 | 912,745 | |
| Days of follow-up (median [IQR]) | 336 [250, 365] | 313 [240, 365] | 0.113 | 365 [352, 365] | 365 [348, 365] | 0.079 |
| Age (median [IQR]) | 41 [29, 54] | 41 [29, 54] | 0.001 | 40 [29, 53] | 40 [29, 53] | 0.002 |
| Age, categories | | | <0.001 | | | <0.001 |
| ≤34 | 83,920 (36.6) | 216,551.4 (36.6) | | 124,643 (37.5) | 342,387.9 (37.5) | |
| 35-49 | 69,937 (30.5) | 180,468.9 (30.5) | | 100,969 (30.4) | 277,356.6 (30.4) | |
| 50-64 | 45,315 (19.8) | 116,933.1 (19.8) | | 79,206 (23.8) | 217,574.8 (23.8) | |
| 65-79 | 21,154 (9.2) | 54,586.8 (9.2) | | 21,026 (6.3) | 57,757.3 (6.3) | |
| ≥80 | 8,760 (3.8) | 22,604.7 (3.8) | | 6,432 (1.9) | 17,668.4 (1.9) | |
| Sex, male | 110,600 (48.3) | 285,397.8 (48.3) | <0.001 | 151,743 (45.7) | 416,830.2 (45.7) | <0.001 |
| Test date period | | | 0.001 | | | <0.001 |
| Sep-Dec 2020 | 59,444 (25.9) | 153,392.3 (25.9) | | 173,455 (52.2) | 476,471.9 (52.2) | |
| Jan-Apr 2021 | 66,558 (29.1) | 171,761.6 (29.1) | | 155,574 (46.8) | 427,394 (46.8) | |
| May-Aug 2021 | 87,942 (38.4) | 226,761.4 (38.4) | | 3,127 (0.9) | 8,528.8 (0.9) | |
| Sep-Dec 2021 | 15,142 (6.6) | 39,229.7 (6.6) | | 120 (0.0) | 350.2 (0.0) | |
| Wave | | | 0.001 | | | 0.001 |
| Alpha | 82,089 (35.8) | 211,682.5 (35.8) | | 213,238 (64.2) | 585,333 (64.1) | |
| Delta | 99,411 (43.4) | 256,513.9 (43.4) | | 1,992 (0.6) | 5,452.7 (0.6) | |
| Wild | 47,586 (20.8) | 122,948.6 (20.8) | | 117,046 (35.2) | 321,959.3 (35.3) | |
| Vaccination status | | | 0.669 | | | 0.080 |
| Not vaccinated | 42,782 (18.7) | 100,551.1 (17.0) | | 42,535 (12.8) | 102,766.1 (11.3) | |
| First dose | 94,845 (41.4) | 116,670.3 (19.7) | | 21,610 (6.5) | 58,537 (6.4) | |
| Two doses | 69,900 (30.5) | 184,339.2 (31.2) | | 109,558 (33.0) | 281,335.1 (30.8) | |
| Three or more (booster) doses | 21,559 (9.4) | 189,584.4 (32.1) | | 158,573 (47.7) | 470,106.8 (51.5) | |
| COVID-19 PCR test | 229,086 (100.0) | 591,145 (100.0) | <0.001 | 332,276 (100.0) | 912,745 (100.0) | <0.001 |

| | | | | | | |
|---|---|---|---|---|---|---|
| Comorbidities | | | | | | |
| Asthma | 16,345 (7.1) | 45,384.1 (7.7) | 0.021 | 50,441 (15.2) | 154,957.6 (17.0) | 0.049 |
| Autoimmune disease | 3,546 (1.5) | 10,333 (1.7) | 0.016 | 7,830 (2.4) | 23,182.4 (2.5) | 0.012 |
| COPD | 4,808 (2.1) | 14,824.3 (2.5) | 0.027 | 3,609 (1.1) | 13,839.1 (1.5) | 0.038 |
| Dementia | 2,488 (1.1) | 4,425.5 (0.7) | 0.035 | 3,098 (0.9) | 6,829.4 (0.7) | 0.020 |
| Diabetes | 16,390 (7.2) | 41,597.3 (7.0) | 0.005 | 21,271 (6.4) | 53,735.3 (5.9) | 0.021 |
| Heart disease | 21,478 (9.4) | 58,345.7 (9.9) | 0.017 | 18,916 (5.7) | 55,286.9 (6.1) | 0.015 |
| Cancer | 12,419 (5.4) | 36,285.4 (6.1) | 0.031 | 12,038 (3.6) | 36,875.5 (4.0) | 0.022 |
| Hypertension | 33,968 (14.8) | 91,441.7 (15.5) | 0.018 | 39,884 (12.0) | 106,583 (11.7) | 0.010 |
| Renal impairment | 7,352 (3.2) | 19,985.6 (3.4) | 0.010 | 10,042 (3.0) | 27,377.5 (3.0) | 0.001 |

SIDIAP = Sistema d'Informació per al Desenvolupament de la Investigació en Atenció Primària, CPRD = Clinical Practice Research Datalink, IQR = interquartile range, with q25 and q75 provided. SMD = standardized mean difference, COPD = Chronic Obstructive Pulmonary Disease

Table S6: Rate Ratios of post-acute COVID-19 symptoms: COVID-19 infection vs first SARS-CoV-2 negative test - 90 days

| Database | Symptom | Events COVID-19 infection | Events first negative test | Rate Ratio [with 95%CI] |
|---|---|---|---|---|
| SIDIAP | Altered smell taste | 329 | 173 | 4.91 [4.08;5.90] |
| | Dyspnoea | 1196 | 2753 | 1.12 [1.05;1.20] |
| | Menstrual problems | 973 | 2404 | 1.04 [0.97;1.12] |
| | Cough | 2947 | 7526 | 1.01 [0.97;1.05] |
| | Headache | 3368 | 8605 | 1.01 [0.97;1.05] |
| | Abdominal pain | 7068 | 18336 | 0.99 [0.97;1.02] |
| | Fatigue malaise | 2676 | 7032 | 0.98 [0.94;1.03] |
| | Tachycardia | 812 | 2143 | 0.98 [0.90;1.06] |
| | Allergy | 2633 | 7038 | 0.97 [0.92;1.01] |
| | Joint pain | 7180 | 19341 | 0.96 [0.93;0.98] |
| | Dizziness | 3039 | 8184 | 0.96 [0.92;1.00] |
| | Chest pain or angina | 2408 | 6490 | 0.96 [0.91;1.00] |
| | Blurred vision | 918 | 2559 | 0.93 [0.86;1.00] |
| | Pins and needles sensation | 713 | 1999 | 0.92 [0.85;1.00] |
| | Anxiety | 3993 | 11356 | 0.91 [0.88;0.94] |
| | Memory issues | 329 | 956 | 0.89 [0.78;1.01] |
| | Tinnitus hearing problems | 1142 | 3370 | 0.87 [0.82;0.93] |
| | Gastrointestinal issues | 4722 | 14156 | 0.86 [0.83;0.89] |
| | Intermittent fever | 1872 | 5693 | 0.85 [0.81;0.89] |
| | Neuralgia | 1729 | 5325 | 0.84 [0.79;0.88] |
| | Sleep disorder | 1905 | 5920 | 0.83 [0.79;0.87] |
| | Cognitive dysfunction | 373 | 1195 | 0.81 [0.72;0.90] |
| | Depression | 1578 | 5188 | 0.78 [0.74;0.83] |
| | Muscle spasms and pain | 213 | 702 | 0.78 [0.67;0.91] |
| CPRD AURUM | Altered smell taste | 471 | 484 | 2.67 [2.35;3.03] |
| | Fatigue malaise | 5600 | 14564 | 1.06 [1.02;1.09] |
| | Menstrual problems | 2219 | 6264 | 0.97 [0.93;1.02] |
| | Allergy | 3534 | 10193 | 0.95 [0.92;0.99] |
| | Memory issues | 341 | 992 | 0.94 [0.84;1.07] |
| | Joint pain | 8964 | 26348 | 0.93 [0.91;0.96] |
| | Tinnitus hearing problems | 2189 | 6476 | 0.93 [0.88;0.97] |
| | Tachycardia | 2812 | 8414 | 0.92 [0.88;0.96] |
| | Neuralgia | 1687 | 5025 | 0.92 [0.87;0.97] |
| | Headache | 6278 | 19004 | 0.91 [0.88;0.93] |
| | Pins and needles sensation | 669 | 2018 | 0.91 [0.83;0.99] |
| | Cognitive dysfunction | 200 | 607 | 0.91 [0.77;1.06] |
| | Abdominal pain | 9069 | 27556 | 0.90 [0.88;0.93] |
| | Dizziness | 2713 | 8247 | 0.90 [0.87;0.94] |
| | Chest pain or angina | 4287 | 13089 | 0.90 [0.87;0.93] |
| | Blurred vision | 363 | 1121 | 0.89 [0.79;1.00] |

| Database | Symptom | Events COVID-19 infection | Events first negative test | Rate Ratio [with 95%CI] |
|---|---|---|---|---|
| | Dyspnoea | 5166 | 16072 | 0.88 [0.86;0.91] |
| | Intermittent fever | 273 | 854 | 0.88 [0.77;1.01] |
| | Cough | 9557 | 30639 | 0.86 [0.84;0.88] |
| | Gastrointestinal issues | 7959 | 25559 | 0.86 [0.83;0.88] |
| | Anxiety | 9887 | 32609 | 0.83 [0.81;0.85] |
| | Sleep disorder | 2501 | 8266 | 0.83 [0.79;0.87] |
| | Muscle spasms and pain | 176 | 580 | 0.83 [0.70;0.99] |
| | Depression | 9489 | 32598 | 0.80 [0.78;0.82] |

SIDIAP = Sistema d'Informació per al Desenvolupament de la Investigació en Atenció Primària, CPRD = Clinical Practice Research Datalink

Table S7: Rate Ratios of post-acute COVID-19 symptoms: COVID-19 infection vs first SARS-CoV-2 negative test - 28 days

| Database | Symptom | Events COVID-19 infection | Events first negative test | Rate Ratio [with 95%CI] |
|---|---|---|---|---|
| SIDIAP | Altered smell taste | 396 | 249 | 4.10 [3.50;4.81] |
| | Dyspnoea | 1783 | 3692 | 1.25 [1.18;1.32] |
| | Menstrual problems | 1280 | 3099 | 1.07 [1.00;1.14] |
| | Tachycardia | 1155 | 2798 | 1.07 [0.99;1.14] |
| | Cough | 3814 | 9394 | 1.05 [1.01;1.09] |
| | Fatigue malaise | 3647 | 9075 | 1.04 [1.00;1.08] |
| | Headache | 4326 | 11204 | 1.00 [0.96;1.03] |
| | Abdominal pain | 9004 | 23735 | 0.98 [0.96;1.00] |
| | Chest pain or angina | 3193 | 8428 | 0.98 [0.94;1.02] |
| | Blurred vision | 1217 | 3218 | 0.98 [0.91;1.04] |
| | Memory issues | 441 | 1188 | 0.96 [0.86;1.07] |
| | Joint pain | 9224 | 24949 | 0.95 [0.93;0.98] |
| | Allergy | 3421 | 9320 | 0.95 [0.91;0.98] |
| | Pins and needles sensation | 922 | 2497 | 0.95 [0.88;1.03] |
| | Dizziness | 3840 | 10492 | 0.94 [0.91;0.98] |
| | Anxiety | 5234 | 14845 | 0.91 [0.88;0.94] |
| | Tinnitus hearing problems | 1530 | 4441 | 0.89 [0.84;0.94] |
| | Gastrointestinal issues | 6074 | 18827 | 0.83 [0.81;0.86] |
| | Neuralgia | 2180 | 6813 | 0.83 [0.79;0.87] |
| | Sleep disorder | 2492 | 7773 | 0.83 [0.79;0.87] |
| | Cognitive dysfunction | 496 | 1592 | 0.80 [0.73;0.89] |
| | Depression | 1992 | 6512 | 0.79 [0.75;0.83] |
| | Intermittent fever | 2303 | 7564 | 0.79 [0.75;0.82] |
| | Muscle spasms and pain | 267 | 897 | 0.77 [0.67;0.88] |
| CPRD AURUM | Altered smell taste | 591 | 568 | 2.86 [2.55;3.21] |
| | Fatigue malaise | 6894 | 17602 | 1.08 [1.05;1.11] |
| | Menstrual problems | 2746 | 7670 | 0.98 [0.94;1.03] |
| | Memory issues | 400 | 1150 | 0.96 [0.85;1.07] |
| | Joint pain | 10527 | 30854 | 0.94 [0.92;0.96] |
| | Dyspnoea | 6536 | 19202 | 0.94 [0.91;0.96] |
| | Tinnitus hearing problems | 2646 | 7734 | 0.94 [0.90;0.98] |
| | Allergy | 4096 | 12006 | 0.94 [0.90;0.97] |
| | Cognitive dysfunction | 230 | 673 | 0.94 [0.81;1.09] |
| | Chest pain or angina | 5411 | 15904 | 0.93 [0.91;0.96] |
| | Tachycardia | 3454 | 10188 | 0.93 [0.90;0.97] |
| | Pins and needles sensation | 833 | 2469 | 0.93 [0.86;1.00] |
| | Abdominal pain | 10996 | 33279 | 0.91 [0.89;0.93] |
| | Neuralgia | 1990 | 6022 | 0.91 [0.86;0.95] |
| | Headache | 7621 | 23225 | 0.90 [0.88;0.92] |
| | Dizziness | 3277 | 10056 | 0.90 [0.86;0.93] |

| Database | Symptom | Events COVID-19 infection | Events first negative test | Rate Ratio [with 95%CI] |
|---|---|---|---|---|
| | Blurred vision | 454 | 1431 | 0.87 [0.78;0.97] |
| | Intermittent fever | 337 | 1066 | 0.87 [0.77;0.98] |
| | Gastrointestinal issues | 9651 | 30651 | 0.86 [0.85;0.88] |
| | Cough | 10980 | 35445 | 0.85 [0.83;0.87] |
| | Anxiety | 11605 | 38132 | 0.84 [0.82;0.85] |
| | Muscle spasms and pain | 215 | 702 | 0.84 [0.72;0.98] |
| | Sleep disorder | 3171 | 10440 | 0.83 [0.80;0.87] |
| | Depression | 10990 | 37888 | 0.80 [0.78;0.81] |

SIDIAP = Sistema d'Informació per al Desenvolupament de la Investigació en Atenció Primària, CPRD = Clinical Practice Research Datalink, CI = confidence intervals
Note: Symptoms with <5 events were not considered.

Table S8: Baseline characteristics: first COVID-19 infection vs re-infections matched 1:3

| | SIDIAP | | | CPRD | | |
|---|---|---|---|---|---|---|
| | **First infection** | **Re-infections** | **SMD** | **First infection** | **Re-infections** | **SMD** |
| N | 155,400 | 55,297 | | 48,574 | 16,916 | |
| Days of follow-up (median [IQR]) | 283 [235, 365] | 270 [215, 365] | 0.102 | 304 [205, 365] | 299 [201, 365] | 0.027 |
| Age (median [IQR]) | 44 [30, 58] | 44 [30, 58] | 0.001 | 47 [33, 59] | 47 [33, 59] | <0.001 |
| Age, categories | | | <0.001 | | | <0.001 |
| ≤34 | 50,450.1 (32.5) | 17,952 (32.5) | | 13,266.2 (27.3) | 4,620 (27.3) | |
| 35-49 | 45,102.2 (29.0) | 16,049 (29.0) | | 13,544.8 (27.9) | 4,717 (27.9) | |
| 50-64 | 34,187 (22.0) | 12,165 (22.0) | | 13,369.6 (27.5) | 4,656 (27.5) | |
| 65-79 | 12,286.5 (7.9) | 4,372 (7.9) | | 5,582.2 (11.5) | 1,944 (11.5) | |
| ≥80 | 13,374.1 (8.6) | 4,759 (8.6) | | 2,811.2 (5.8) | 979 (5.8) | |
| Sex, male | 53,139.6 (34.2) | 18,909 (34.2) | <0.001 | 18,575.6 (38.2) | 6,469 (38.2) | <0.001 |
| Test date period | | | 0.001 | | | 0.004 |
| Sep-Dec 2020 | 31,107 (20.0) | 11,069 (20.0) | | 11,954 (24.6) | 4,163 (24.6) | |
| Jan-Apr 2021 | 47,742 (30.7) | 16,985 (30.7) | | 20,852.2 (42.9) | 7,228 (42.7) | |
| May-Aug 2021 | 64,410.7 (41.4) | 22,907 (41.4) | | 14,450.7 (29.7) | 5,064 (29.9) | |
| Sep-Dec 2021 | 12,140.4 (7.8) | 4,336 (7.8) | | 1,317.1 (2.7) | 461 (2.7) | |
| Wave | | | 0.002 | | | 0.001 |
| Alpha | 57,089.9 (36.7) | 20,344 (36.8) | | 26,036.2 (53.6) | 9,071 (53.6) | |
| Delta | 73,833.5 (47.5) | 26,275 (47.5) | | 13,709.4 (28.2) | 4,770 (28.2) | |
| Wild | 24,476.6 (15.8) | 8,678 (15.7) | | 8,828.4 (18.2) | 3,075 (18.2) | |
| Vaccination status | | | 0.128 | | | 0.056 |
| Not vaccinated | 30,977.2 (19.9) | 9,718 (17.6) | | 8,605.4 (17.7) | 2,673 (15.8) | |
| First dose | 53,731.1 (34.6) | 22,147 (40.1) | | 12,033.9 (24.8) | 4,319 (25.5) | |
| Two doses | 40,876 (26.3) | 14,506 (26.2) | | 14,421.1 (29.7) | 5,268 (31.1) | |
| Three or more (booster) doses | 29,815.7 (19.2) | 8,926 (16.1) | | 13,513.7 (27.8) | 4,656 (27.5) | |
| COVID-19 PCR test | 61,362.4 (39.5) | 21,835 (39.5) | <0.001 | 14,501 (29.9) | 5,050 (29.9) | <0.001 |

| | | | | | | |
|---|---|---|---|---|---|---|
| Comorbidities | | | | | | |
| Asthma | 11,929.2 (7.7) | 5,281 (9.6) | 0.067 | 9,041.3 (18.6) | 4,025 (23.8) | 0.127 |
| Autoimmune disease | 3,348.4 (2.2) | 1,415 (2.6) | 0.027 | 1,695.6 (3.5) | 723 (4.3) | 0.041 |
| COPD | 4,520.3 (2.9) | 2,086 (3.8) | 0.048 | 1,936.3 (4.0) | 889 (5.3) | 0.060 |
| Dementia | 4,052 (2.6) | 2,594 (4.7) | 0.111 | 807.8 (1.7) | 376 (2.2) | 0.041 |
| Diabetes | 13,948.8 (9.0) | 6,004 (10.9) | 0.063 | 4,967.2 (10.2) | 2,136 (12.6) | 0.076 |
| Heart disease | 19,341.3 (12.4) | 8,590 (15.5) | 0.089 | 5,346.2 (11.0) | 2,306 (13.6) | 0.080 |
| Cancer | 11,583.5 (7.5) | 4,808 (8.7) | 0.046 | 3,183.5 (6.6) | 1,116 (6.6) | 0.002 |
| Hypertension | 29,947.3 (19.3) | 11,971 (21.6) | 0.059 | 9,355.3 (19.3) | 3,582 (21.2) | 0.048 |
| Renal impairment | 8,321.2 (5.4) | 3,849 (7.0) | 0.067 | 3,265.8 (6.7) | 1,492 (8.8) | 0.078 |

SIDIAP = Sistema d'Informació per al Desenvolupament de la Investigació en Atenció Primària, CPRD = Clinical Practice Research Datalink, IQR = interquartile range, with q25 and q75 provided. COPD = Chronic Obstructive Pulmonary Disease, SMD = Standardized Mean Difference

Figure S3: Rate Ratios of post-acute COVID-19 symptoms: COVID-19 infection vs first SARS-CoV-2 negative test - 28 days

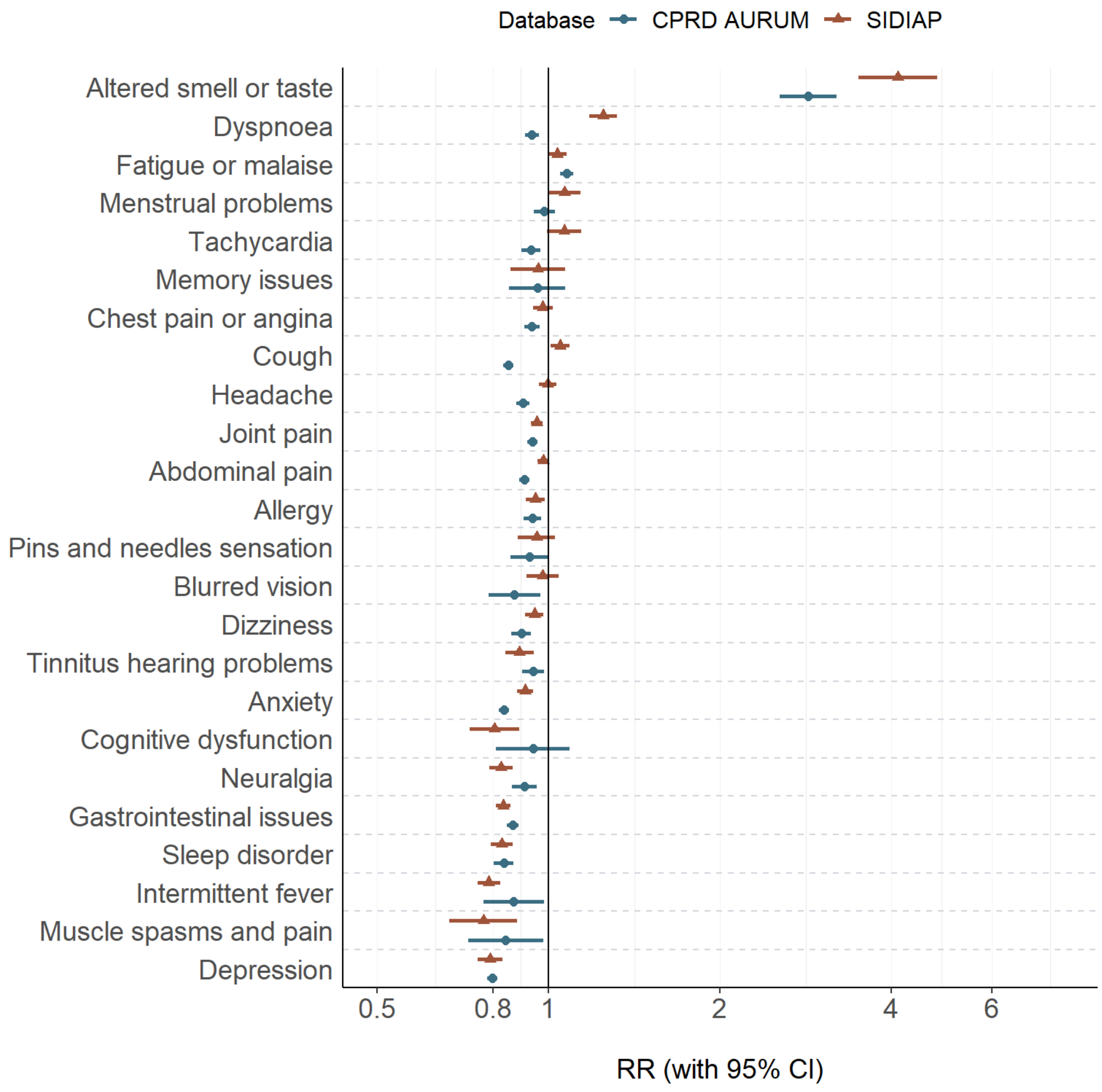


SIDIAP = Sistema d'Informació per al Desenvolupament de la Investigació en Atenció Primària, CPRD = Clinical Practice Research Datalink, RR = Rate Ratio, 95% CI = 95% confidence intervals
Table S7 provides the sample size, rate ratios and 95%CI for post-acute COVID-19 symptoms for COVID-19 infection vs first SARS-CoV-2 negative test at >= 28 days.

Table S9: Rate ratios of post-acute COVID-19 symptoms, COVID-19 infection vs all SARS-CoV-2 negative tests - 90 days

| Database | Symptom | Events COVID-19 infection | Events first negative test | Rate Ratio [with 95%CI] |
|---|---|---|---|---|
| SIDIAP | Altered smell taste | 329 | 173 | 4.91 [4.08;5.90] |
| | Dyspnoea | 1196 | 2753 | 1.12 [1.05;1.20] |
| | Menstrual problems | 973 | 2404 | 1.04 [0.97;1.12] |
| | Cough | 2947 | 7526 | 1.01 [0.97;1.05] |
| | Headache | 3368 | 8605 | 1.01 [0.97;1.05] |
| | Abdominal pain | 7068 | 18336 | 0.99 [0.97;1.02] |
| | Fatigue malaise | 2676 | 7032 | 0.98 [0.94;1.03] |
| | Tachycardia | 812 | 2143 | 0.98 [0.90;1.06] |
| | Allergy | 2633 | 7038 | 0.97 [0.92;1.01] |
| | Joint pain | 7180 | 19341 | 0.96 [0.93;0.98] |
| | Dizziness | 3039 | 8184 | 0.96 [0.92;1.00] |
| | Chest pain or angina | 2408 | 6490 | 0.96 [0.91;1.00] |
| | Blurred vision | 918 | 2559 | 0.93 [0.86;1.00] |
| | Pins and needles sensation | 713 | 1999 | 0.92 [0.85;1.00] |
| | Anxiety | 3993 | 11356 | 0.91 [0.88;0.94] |
| | Memory issues | 329 | 956 | 0.89 [0.78;1.01] |
| | Tinnitus hearing problems | 1142 | 3370 | 0.87 [0.82;0.93] |
| | Gastrointestinal issues | 4722 | 14156 | 0.86 [0.83;0.89] |
| | Intermittent fever | 1872 | 5693 | 0.85 [0.81;0.89] |
| | Neuralgia | 1729 | 5325 | 0.84 [0.79;0.88] |
| | Sleep disorder | 1905 | 5920 | 0.83 [0.79;0.87] |
| | Cognitive dysfunction | 373 | 1195 | 0.81 [0.72;0.90] |
| | Depression | 1578 | 5188 | 0.78 [0.74;0.83] |
| | Muscle spasms and pain | 213 | 702 | 0.78 [0.67;0.91] |
| CPRD AURUM | Altered smell taste | 471 | 484 | 2.67 [2.35;3.03] |
| | Fatigue malaise | 5600 | 14564 | 1.06 [1.02;1.09] |
| | Menstrual problems | 2219 | 6264 | 0.97 [0.93;1.02] |
| | Allergy | 3534 | 10193 | 0.95 [0.92;0.99] |
| | Memory issues | 341 | 992 | 0.94 [0.84;1.07] |
| | Joint pain | 8964 | 26348 | 0.93 [0.91;0.96] |
| | Tinnitus hearing problems | 2189 | 6476 | 0.93 [0.88;0.97] |
| | Tachycardia | 2812 | 8414 | 0.92 [0.88;0.96] |
| | Neuralgia | 1687 | 5025 | 0.92 [0.87;0.97] |
| | Headache | 6278 | 19004 | 0.91 [0.88;0.93] |
| | Pins and needles sensation | 669 | 2018 | 0.91 [0.83;0.99] |
| | Cognitive dysfunction | 200 | 607 | 0.91 [0.77;1.06] |
| | Abdominal pain | 9069 | 27556 | 0.90 [0.88;0.93] |
| | Dizziness | 2713 | 8247 | 0.90 [0.87;0.94] |
| | Chest pain or angina | 4287 | 13089 | 0.90 [0.87;0.93] |

| Database | Symptom | Events COVID-19 infection | Events first negative test | Rate Ratio [with 95%CI] |
|---|---|---|---|---|
| | Blurred vision | 363 | 1121 | 0.89 [0.79;1.00] |
| | Dyspnoea | 5166 | 16072 | 0.88 [0.86;0.91] |
| | Intermittent fever | 273 | 854 | 0.88 [0.77;1.01] |
| | Cough | 9557 | 30639 | 0.86 [0.84;0.88] |
| | Gastrointestinal issues | 7959 | 25559 | 0.86 [0.83;0.88] |
| | Anxiety | 9887 | 32609 | 0.83 [0.81;0.85] |
| | Sleep disorder | 2501 | 8266 | 0.83 [0.79;0.87] |
| | Muscle spasms and pain | 176 | 580 | 0.83 [0.70;0.99] |
| | Depression | 9489 | 32598 | 0.80 [0.78;0.82] |

SIDIAP = Sistema d'Informació per al Desenvolupament de la Investigació en Atenció Primària, CPRD = Clinical Practice Research Datalink, CI = confidence intervals

Figure S4: Rate Ratios of post-acute COVID-19 symptoms, COVID-19 infection vs all SARS-CoV-2 negative tests - 90 days

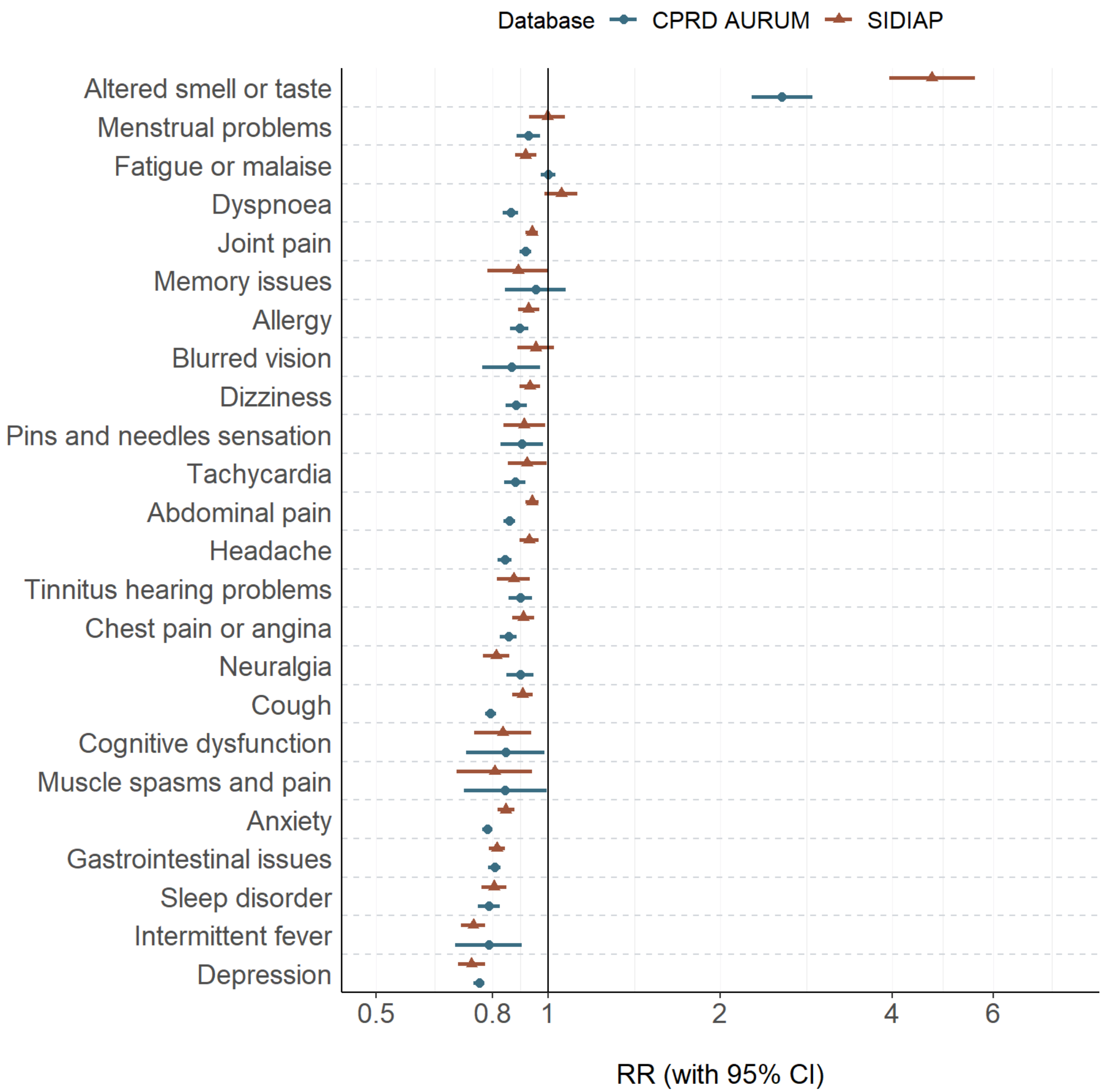


SIDIAP = Sistema d'Informació per al Desenvolupament de la Investigació en Atenció Primària, CPRD = Clinical Practice Research Datalink, RR = Rate ratio, 95% CI = 95% confidence intervals
Table S9 provides the sample size, rate ratios and 95%CI for post-acute COVID-19 symptoms for COVID-19 infection vs all SARS-CoV-2 negative test at >= 90 days.

Table S10: Rate ratio of post-acute COVID-19 symptoms, COVID-19 infection vs all SARS-CoV-2 negative tests - 28 days

| Database | Symptom | Events COVID-19 infection | Events first negative test | Rate Ratio [with 95%CI] |
|---|---|---|---|---|
| SIDIAP | Altered smell taste | 396 | 249 | 4.10 [3.50;4.81] |
| | Dyspnoea | 1783 | 3692 | 1.25 [1.18;1.32] |
| | Menstrual problems | 1280 | 3099 | 1.07 [1.00;1.14] |
| | Tachycardia | 1155 | 2798 | 1.07 [0.99;1.14] |
| | Cough | 3814 | 9394 | 1.05 [1.01;1.09] |
| | Fatigue malaise | 3647 | 9075 | 1.04 [1.00;1.08] |
| | Headache | 4326 | 11204 | 1.00 [0.96;1.03] |
| | Abdominal pain | 9004 | 23735 | 0.98 [0.96;1.00] |
| | Chest pain or angina | 3193 | 8428 | 0.98 [0.94;1.02] |
| | Blurred vision | 1217 | 3218 | 0.98 [0.91;1.04] |
| | Memory issues | 441 | 1188 | 0.96 [0.86;1.07] |
| | Joint pain | 9224 | 24949 | 0.95 [0.93;0.98] |
| | Allergy | 3421 | 9320 | 0.95 [0.91;0.98] |
| | Pins and needles sensation | 922 | 2497 | 0.95 [0.88;1.03] |
| | Dizziness | 3840 | 10492 | 0.94 [0.91;0.98] |
| | Anxiety | 5234 | 14845 | 0.91 [0.88;0.94] |
| | Tinnitus hearing problems | 1530 | 4441 | 0.89 [0.84;0.94] |
| | Gastrointestinal issues | 6074 | 18827 | 0.83 [0.81;0.86] |
| | Neuralgia | 2180 | 6813 | 0.83 [0.79;0.87] |
| | Sleep disorder | 2492 | 7773 | 0.83 [0.79;0.87] |
| | Cognitive dysfunction | 496 | 1592 | 0.80 [0.73;0.89] |
| | Depression | 1992 | 6512 | 0.79 [0.75;0.83] |
| | Intermittent fever | 2303 | 7564 | 0.79 [0.75;0.82] |
| | Muscle spasms and pain | 267 | 897 | 0.77 [0.67;0.88] |
| CPRD AURUM | Altered smell taste | 591 | 568 | 2.86 [2.55;3.21] |
| | Fatigue malaise | 6894 | 17602 | 1.08 [1.05;1.11] |
| | Menstrual problems | 2746 | 7670 | 0.98 [0.94;1.03] |
| | Memory issues | 400 | 1150 | 0.96 [0.85;1.07] |
| | Joint pain | 10527 | 30854 | 0.94 [0.92;0.96] |
| | Dyspnoea | 6536 | 19202 | 0.94 [0.91;0.96] |
| | Tinnitus hearing problems | 2646 | 7734 | 0.94 [0.90;0.98] |
| | Allergy | 4096 | 12006 | 0.94 [0.90;0.97] |
| | Cognitive dysfunction | 230 | 673 | 0.94 [0.81;1.09] |
| | Chest pain or angina | 5411 | 15904 | 0.93 [0.91;0.96] |
| | Tachycardia | 3454 | 10188 | 0.93 [0.90;0.97] |
| | Pins and needles sensation | 833 | 2469 | 0.93 [0.86;1.00] |
| | Abdominal pain | 10996 | 33279 | 0.91 [0.89;0.93] |
| | Neuralgia | 1990 | 6022 | 0.91 [0.86;0.95] |

| Database | Symptom | Events COVID-19 infection | Events first negative test | Rate Ratio [with 95%CI] |
|---|---|---|---|---|
| | Headache | 7621 | 23225 | 0.90 [0.88;0.92] |
| | Dizziness | 3277 | 10056 | 0.90 [0.86;0.93] |
| | Blurred vision | 454 | 1431 | 0.87 [0.78;0.97] |
| | Intermittent fever | 337 | 1066 | 0.87 [0.77;0.98] |
| | Gastrointestinal issues | 9651 | 30651 | 0.86 [0.85;0.88] |
| | Cough | 10980 | 35445 | 0.85 [0.83;0.87] |
| | Anxiety | 11605 | 38132 | 0.84 [0.82;0.85] |
| | Muscle spasms and pain | 215 | 702 | 0.84 [0.72;0.98] |
| | Sleep disorder | 3171 | 10440 | 0.83 [0.80;0.87] |
| | Depression | 10990 | 37888 | 0.80 [0.78;0.81] |

SIDIAP = Sistema d'Informació per al Desenvolupament de la Investigació en Atenció Primària, CPRD = Clinical Practice Research Datalink, CI = confidence intervals

Figure S5: Rate ratios of post-acute COVID-19 symptoms, COVID-19 infection vs all SARS-CoV-2 negative tests - 28 days

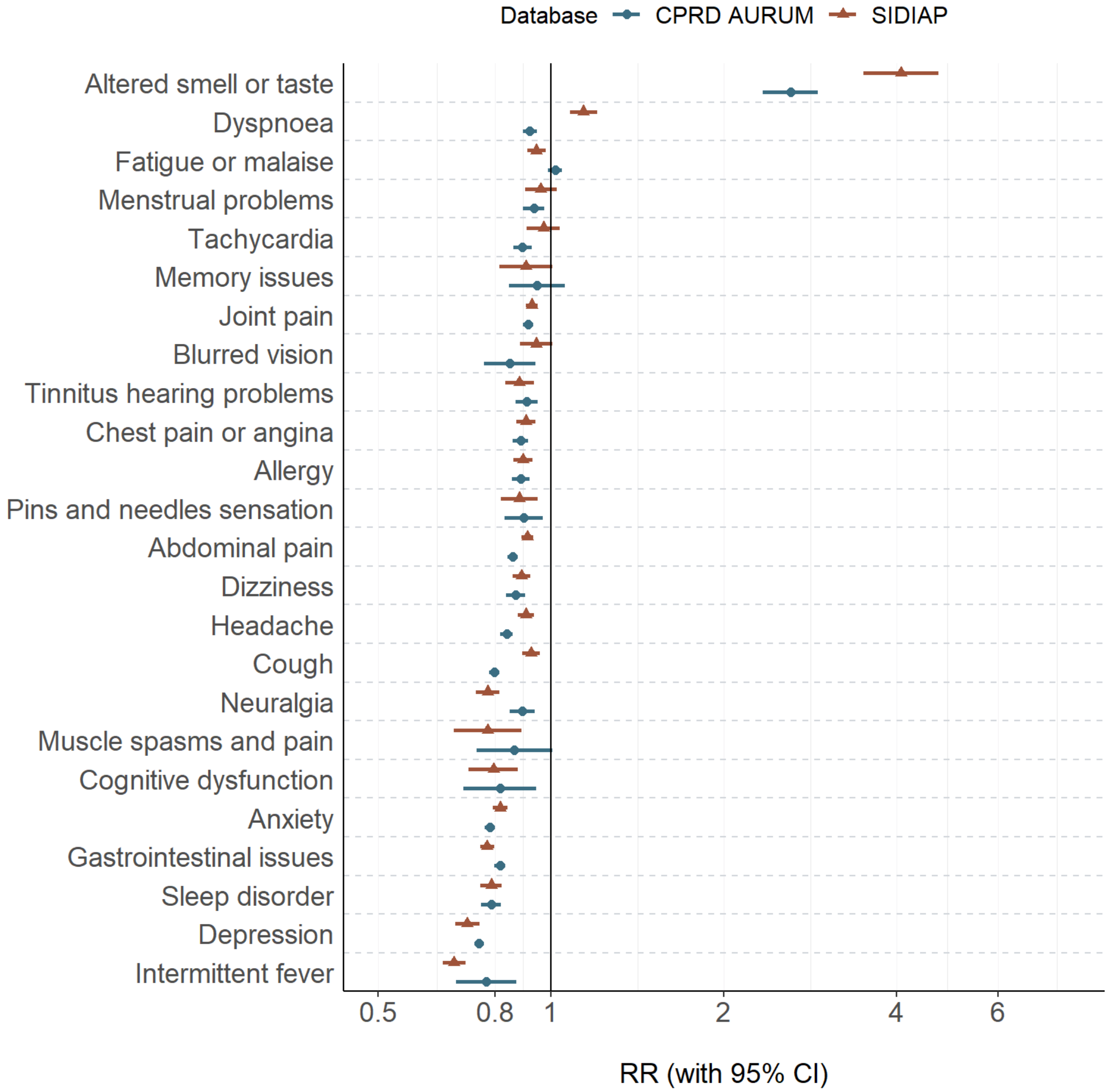


SIDIAP = Sistema d'Informació per al Desenvolupament de la Investigació en Atenció Primària, CPRD = Clinical Practice Research Datalink, RR = Rate ratios, 95% CI = 95% confidence intervals
Table S10 provides the sample size, rate ratios and 95%CI for post-acute COVID-19 symptoms for COVID-19 infection vs all SARS-CoV-2 negative test at >= 28 days.

Table S11: Rate ratio of post-acute COVID-19 symptoms, first infection vs re-infection - 90 days

| Database | Symptom | Events re-infection | Events first infection | Rate Ratio [with 95%CI] |
|---|---|---|---|---|
| SIDIAP | Altered smell taste | 65 | 135 | 1.35 [1.01;1.82] |
| | Allergy | 1116 | 2233 | 1.40 [1.31;1.51] |
| | Tachycardia | 337 | 667 | 1.42 [1.25;1.62] |
| | Sleep disorder | 846 | 1670 | 1.42 [1.31;1.55] |
| | Joint pain | 3026 | 5975 | 1.42 [1.36;1.49] |
| | Menstrual problems | 477 | 936 | 1.43 [1.28;1.60] |
| | Dizziness | 1335 | 2629 | 1.43 [1.34;1.52] |
| | Memory issues | 140 | 274 | 1.44 [1.17;1.76] |
| | Anxiety | 1800 | 3504 | 1.44 [1.37;1.53] |
| | Tinnitus hearing problems | 519 | 997 | 1.46 [1.32;1.63] |
| | Abdominal pain | 3055 | 5853 | 1.47 [1.41;1.53] |
| | Muscle spasms and pain | 107 | 203 | 1.48 [1.17;1.87] |
| | Gastrointestinal issues | 2252 | 4275 | 1.48 [1.41;1.56] |
| | Depression | 831 | 1554 | 1.50 [1.38;1.63] |
| | Headache | 1480 | 2777 | 1.50 [1.41;1.59] |
| | Neuralgia | 841 | 1567 | 1.51 [1.39;1.64] |
| | Fatigue malaise | 1249 | 2310 | 1.52 [1.42;1.63] |
| | Chest pain or angina | 1103 | 2025 | 1.53 [1.42;1.65] |
| | Blurred vision | 439 | 803 | 1.54 [1.37;1.73] |
| | Cognitive dysfunction | 236 | 429 | 1.55 [1.32;1.81] |
| | Dyspnoea | 548 | 961 | 1.60 [1.44;1.78] |
| | Cough | 1458 | 2529 | 1.62 [1.52;1.73] |
| | Pins and needles sensation | 320 | 550 | 1.64 [1.43;1.88] |
| | Intermittent fever | 1103 | 1813 | 1.71 [1.59;1.84] |
| CPRD AURUM | Muscle spasms and pain | 15 | 44 | 0.98 [0.54;1.76] |
| | Menstrual problems | 98 | 255 | 1.10 [0.87;1.39] |
| | Dyspnoea | 592 | 1390 | 1.22 [1.11;1.34] |
| | Neuralgia | 120 | 267 | 1.29 [1.04;1.60] |
| | Dizziness | 212 | 458 | 1.33 [1.13;1.56] |
| | Anxiety | 671 | 1453 | 1.33 [1.21;1.45] |
| | Joint pain | 651 | 1386 | 1.35 [1.23;1.48] |
| | Tinnitus hearing problems | 177 | 375 | 1.36 [1.13;1.62] |
| | Tachycardia | 259 | 534 | 1.39 [1.20;1.61] |
| | Headache | 438 | 901 | 1.40 [1.25;1.56] |
| | Altered smell taste | 24 | 49 | 1.41 [0.86;2.29] |
| | Depression | 736 | 1504 | 1.41 [1.29;1.53] |
| | Abdominal pain | 662 | 1326 | 1.43 [1.31;1.57] |
| | Cough | 968 | 1915 | 1.45 [1.35;1.56] |
| | Gastrointestinal issues | 719 | 1412 | 1.46 [1.34;1.60] |
| | Sleep disorder | 251 | 489 | 1.47 [1.27;1.71] |
| | Blurred vision | 32 | 62 | 1.48 [0.97;2.27] |

| Database | Symptom | Events re-infection | Events first infection | Rate Ratio [with 95%CI] |
|---|---|---|---|---|
| | Fatigue malaise | 459 | 884 | 1.49 [1.33;1.67] |
| | Memory issues | 53 | 101 | 1.51 [1.08;2.10] |
| | Chest pain or angina | 385 | 728 | 1.52 [1.34;1.72] |
| | Allergy | 249 | 418 | 1.71 [1.46;2.00] |
| | Pins and needles sensation | 62 | 94 | 1.89 [1.38;2.61] |
| | Intermittent fever | 46 | 61 | 2.17 [1.48;3.17] |
| | Cognitive dysfunction | 33 | 42 | 2.26 [1.43;3.56] |

SIDIAP = Sistema d'Informació per al Desenvolupament de la Investigació en Atenció Primària, CPRD = Clinical Practice Research Datalink, CI = confidence intervals
Note: Symptoms with <5 events were not considered.

Table S12: Rate ratios of post-acute COVID-19 symptoms, first infection vs re-infection - 28 days

| Database | Symptom | Events re-infection | Events first infection | Rate Ratio [with 95%CI] |
|---|---|---|---|---|
| SIDIAP | Memory issues | 213 | 422 | 1.42 [1.20;1.67] |
| | Menstrual problems | 648 | 1252 | 1.45 [1.32;1.60] |
| | Tachycardia | 478 | 913 | 1.47 [1.32;1.64] |
| | Joint pain | 4165 | 7893 | 1.48 [1.43;1.54] |
| | Dizziness | 1878 | 3547 | 1.49 [1.41;1.57] |
| | Gastrointestinal issues | 3104 | 5808 | 1.50 [1.44;1.57] |
| | Tinnitus hearing problems | 710 | 1324 | 1.51 [1.38;1.65] |
| | Anxiety | 2548 | 4716 | 1.52 [1.45;1.59] |
| | Sleep disorder | 1221 | 2241 | 1.53 [1.43;1.64] |
| | Blurred vision | 620 | 1128 | 1.54 [1.40;1.70] |
| | Allergy | 1635 | 2989 | 1.54 [1.45;1.63] |
| | Fatigue malaise | 1763 | 3217 | 1.54 [1.45;1.63] |
| | Altered smell taste | 90 | 163 | 1.55 [1.20;2.01] |
| | Muscle spasms and pain | 146 | 265 | 1.55 [1.27;1.89] |
| | Cognitive dysfunction | 324 | 586 | 1.55 [1.36;1.78] |
| | Neuralgia | 1165 | 2106 | 1.55 [1.45;1.67] |
| | Abdominal pain | 4284 | 7748 | 1.55 [1.50;1.61] |
| | Headache | 1993 | 3569 | 1.57 [1.49;1.66] |
| | Pins and needles sensation | 438 | 779 | 1.58 [1.41;1.78] |
| | Depression | 1126 | 1986 | 1.59 [1.48;1.71] |
| | Dyspnoea | 814 | 1425 | 1.61 [1.47;1.75] |
| | Chest pain or angina | 1579 | 2723 | 1.63 [1.53;1.73] |
| | Cough | 1969 | 3300 | 1.68 [1.59;1.77] |
| | Intermittent fever | 1560 | 2420 | 1.81 [1.70;1.93] |
| CPRD AURUM | Muscle spasms and pain | 17 | 52 | 0.94 [0.54;1.62] |
| | Menstrual problems | 137 | 349 | 1.13 [0.93;1.37] |
| | Blurred vision | 46 | 108 | 1.22 [0.87;1.73] |
| | Neuralgia | 148 | 341 | 1.25 [1.03;1.51] |
| | Dizziness | 278 | 613 | 1.30 [1.13;1.50] |
| | Dyspnoea | 837 | 1822 | 1.32 [1.22;1.43] |
| | Joint pain | 823 | 1759 | 1.34 [1.24;1.46] |
| | Tachycardia | 345 | 730 | 1.36 [1.20;1.54] |
| | Anxiety | 834 | 1758 | 1.36 [1.26;1.48] |
| | Tinnitus hearing problems | 231 | 484 | 1.37 [1.17;1.60] |
| | Headache | 567 | 1163 | 1.40 [1.27;1.55] |
| | Gastrointestinal issues | 944 | 1923 | 1.41 [1.31;1.52] |
| | Cough | 1201 | 2387 | 1.44 [1.35;1.55] |
| | Abdominal pain | 856 | 1693 | 1.45 [1.34;1.57] |
| | Depression | 902 | 1721 | 1.50 [1.39;1.63] |
| | Sleep disorder | 329 | 626 | 1.51 [1.32;1.72] |
| | Chest pain or angina | 528 | 994 | 1.53 [1.37;1.69] |

| Database | Symptom | Events re-infection | Events first infection | Rate Ratio [with 95%CI] |
|---|---|---|---|---|
| | Memory issues | 72 | 132 | 1.57 [1.18;2.09] |
| | Allergy | 328 | 591 | 1.59 [1.39;1.82] |
| | Fatigue malaise | 615 | 1089 | 1.62 [1.47;1.79] |
| | Altered smell taste | 36 | 60 | 1.72 [1.14;2.60] |
| | Pins and needles sensation | 82 | 122 | 1.93 [1.46;2.55] |
| | Intermittent fever | 61 | 77 | 2.27 [1.63;3.18] |
| | Cognitive dysfunction | 42 | 48 | 2.51 [1.66;3.80] |

SIDIAP = Sistema d'Informació per al Desenvolupament de la Investigació en Atenció Primària, CPRD = Clinical Practice Research Datalink, CI = confidence intervals
Note: Symptoms with <5 events were not considered.

Figure S6: Rate ratio of post-acute COVID-19 symptoms, first infection vs re-infection - 28 days

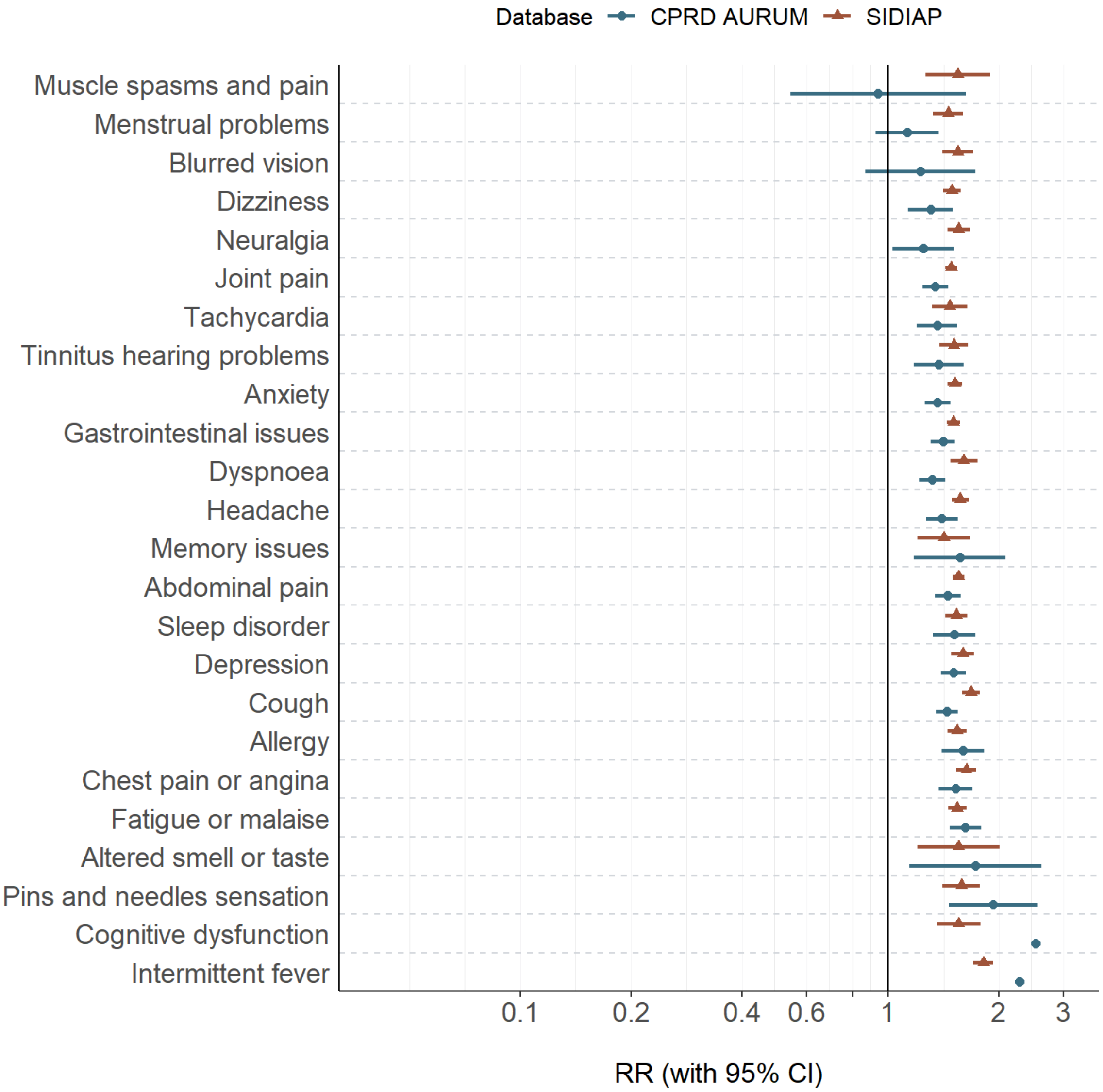


SIDIAP = Sistema d'Informació per al Desenvolupament de la Investigació en Atenció Primària, CPRD = Clinical Practice Research Datalink, RR = Rate ratio, 95% CI = 95% confidence intervals
Table S12 provides the sample size, rate ratios and 95%CI for post-acute COVID-19 symptoms for first SARS-CoV-2 infection vs reinfection at >= 28 days.
