# Supplementary material for: The burden of post-acute COVID-19 symptoms in a multinational network cohort analysis
Source: Nat Commun. 2023 Nov 17;14:7449. doi: 10.1038/s41467-023-42726-0 (PMC10656441; doi:10.1038/s41467-023-42726-0)
Supplement: Supplementary file 3 — Reporting Summary [file 41467_2023_42726_MOESM3_ESM.pdf]

nature portfolio

Corresponding author(s): Prof. Daniel Prieto-Alhambra

Last updated by author(s): Oct 9, 2023

Reporting Summary

Nature Portfolio wishes to improve the reproducibility of the work that we publish. This form provides structure for consistency and transparency in reporting. For further information on Nature Portfolio policies, see our Editorial Policies and the Editorial Policy Checklist.

Statistics

For all statistical analyses, confirm that the following items are present in the figure legend, table legend, main text, or Methods section.

n/a	Confirmed	
☐	☒	The exact sample size (*n*) for each experimental group/condition, given as a discrete number and unit of measurement
☐	☒	A statement on whether measurements were taken from distinct samples or whether the same sample was measured repeatedly
☐	☒	The statistical test(s) used AND whether they are one- or two-sided<br>*Only common tests should be described solely by name; describe more complex techniques in the Methods section.*
☐	☒	A description of all covariates tested
☐	☒	A description of any assumptions or corrections, such as tests of normality and adjustment for multiple comparisons
☐	☒	A full description of the statistical parameters including central tendency (e.g. means) or other basic estimates (e.g. regression coefficient) AND variation (e.g. standard deviation) or associated estimates of uncertainty (e.g. confidence intervals)
☒	☐	For null hypothesis testing, the test statistic (e.g. *F*, *t*, *r*) with confidence intervals, effect sizes, degrees of freedom and *P* value noted<br>*Give P values as exact values whenever suitable.*
☒	☐	For Bayesian analysis, information on the choice of priors and Markov chain Monte Carlo settings
☒	☐	For hierarchical and complex designs, identification of the appropriate level for tests and full reporting of outcomes
☒	☐	Estimates of effect sizes (e.g. Cohen's *d*, Pearson's *r*), indicating how they were calculated

Our web collection on statistics for biologists contains articles on many of the points above.

Software and code

Policy information about availability of computer code

Data collection	No software was used for data collection, but datasets were directly received from SIDIAP and CPRD, respectively.
Data analysis	Analyses were conducted in R (version 4.3.1). All analytical code is available at https://github.com/oxford-pharmacoepi/LongCOVIDWP1A.

For manuscripts utilizing custom algorithms or software that are central to the research but not yet described in published literature, software must be made available to editors and reviewers. We strongly encourage code deposition in a community repository (e.g. GitHub). See the Nature Portfolio guidelines for submitting code & software for further information.

Data

Policy information about availability of data

All manuscripts must include a data availability statement. This statement should provide the following information, where applicable:

- Accession codes, unique identifiers, or web links for publicly available datasets
- A description of any restrictions on data availability
- For clinical datasets or third party data, please ensure that the statement adheres to our policy

CPRD data were obtained under the CPRD multi-study license held by the University of Oxford after Research Data Governance (RDG) approval. Direct data sharing is not allowed.
Following current European and national law, SIDIAP data are only available for researchers participating in this study. However, researchers from public institutions can request data from SIDIAP if they comply with certain requirements. Further information is available online (https://www.sidiap.org/index.php/en/solicituds-en)

or by contacting SIDIAP (sidiap@idiapjgol.org).

Research involving human participants, their data, or biological material

Policy information about studies with human participants or human data. See also policy information about sex, gender (identity/presentation), and sexual orientation and race, ethnicity and racism.

Reporting on sex and gender	We report sex/gender in this manuscript based on the sex/gender recorded by general practitioners in primary care routinely-collected data. We use the term "sex" in the manuscript.
Reporting on race, ethnicity, or other socially relevant groupings	Not applicable as no reporting of ethnicity or race in this study
Population characteristics	We describe and compare baseline characteristics including age groups [≤34, 35-49, 50-64, 65-79 and ≥80], sex, calendar time [trimester, waves], COVID-19 vaccine status [unvaccinated and number of vaccine doses received], and co-morbidities
Recruitment	All people in the database were eligible for inclusion to the respective cohorts if they were aged ≥18 years with ≥180 days of data visibility available before the index date [date of COVID-19 negative test]. Individuals with an influenza clinical diagnosis or positive test result for influenza 42 days before or on the index date were excluded. To ensure sufficient follow-up to assess post-acute COVID-19 symptoms and reduce survival bias, we only included individuals with ≥120 days of follow-up, i.e., with an index date ≥120 days before the end of data availability.
Ethics oversight	The study was approved by the relevant Institution Review Boards: the CPRD's Research Data Governance Process (Protocol No 21_000557), the Clinical Research Ethics committee of Fundació Institut Universitari per a la recerca a l'Atenció Primària de Salut Jordi Gol i Gurina (IDIAPJGol) (approval number 4R22/133). No informed consent of individuals was required as the study only used secondary data.

Note that full information on the approval of the study protocol must also be provided in the manuscript.

Field-specific reporting

Please select the one below that is the best fit for your research. If you are not sure, read the appropriate sections before making your selection.

☒ Life sciences ☐ Behavioural & social sciences ☐ Ecological, evolutionary & environmental sciences

For a reference copy of the document with all sections, see nature.com/documents/nr-reporting-summary-flat.pdf

Life sciences study design

All studies must disclose on these points even when the disclosure is negative.

Sample size	No sample size has been calculates for this purely observational study. All people in SIDIAP and CPRD who met the eligibility criteria for inclusion into ant of the respective study cohorts were included. The study inclusion process with number of people in- and excluded with each inclusion criterion is illustrated in Figure 1.
Data exclusions	No data was excluded, but the whole SIDIAP and CPRD datacuts were used. From this, the study cohorts were derived by applying in-/exclusion criteria, as detailed in the Methods section and illustrated in the study inclusion flowchart.
Replication	While no "replication" of the study has been conducted, we conducted the exact same study in 2 different databases, from 2 countries. Findings were largely consistent across databases.
Randomization	This is a purely observational study using routinely-collected electronic health data. Hence, no randomization could be conducted.
Blinding	This is a purely observational study using routinely-collected electronic health data. Hence, no blinding could be conducted.

Reporting for specific materials, systems and methods

We require information from authors about some types of materials, experimental systems and methods used in many studies. Here, indicate whether each material, system or method listed is relevant to your study. If you are not sure if a list item applies to your research, read the appropriate section before selecting a response.

Materials & experimental systems

n/a	Involved in the study
☒	☐ Antibodies
☒	☐ Eukaryotic cell lines
☒	☐ Palaeontology and archaeology
☒	☐ Animals and other organisms
☒	☐ Clinical data
☒	☐ Dual use research of concern
☒	☐ Plants

Methods

n/a	Involved in the study
☒	☐ ChIP-seq
☒	☐ Flow cytometry
☒	☐ MRI-based neuroimaging

Plants

Seed stocks	NA
Novel plant genotypes	NA
Authentication	NA
